# Effects of biological agents on rhizosphere microecological environment and nutrient availability for rice

**DOI:** 10.3389/fmicb.2024.1447527

**Published:** 2025-01-08

**Authors:** Hang Zhou, Kaibo Yu, Lingli Nie, Lang Liu, Jianqun Zhou, Kunlun Wu, Honghong Ye, Zhaohui Wu

**Affiliations:** ^1^National Key Laboratory of Hybrid Rice, Hunan Hybrid Rice Research Center, Changsha, China; ^2^School of Tropical Agriculture and Forestry, Hainan University, Haikou, China; ^3^National Saline-Alkali Tolerant Rice Technology Innovation Center, Changsha, China; ^4^Heilongjiang Jiaze Complex Enzyme Technology Research Center, Harbin, China; ^5^Hunan Institute of Agricultural Information and Engineering, Changsha, China

**Keywords:** rhizosphere microorganisms, rice, biological agents, yield, metabolome

## Abstract

As the world’s population grows, pursuing sustainable agricultural production techniques to increase crop yields is critical to ensuring global food security. The development and application of biological agents is of great significance in promoting the sustainable development of agriculture. This study aimed to investigate the role of JZ (compound microbial agent) and MZ (biological agent made from plant materials) in improving the rhizosphere microecological environment and nutrient availability for rice. This study found that JZ enriched *Cyanobacteria* with biological nitrogen fixation functions; spraying MZ can enrich some beneficial microbiota, such as *Bradyrhizobium*, playing a role in symbiotic nitrogen fixation. Meanwhile, JZ and MZ were found to affect rhizosphere soil metabolism and improve potassium and nitrogen availability. JZ may promote the degradation of fungicides in the rhizosphere soil environment. Overall, applying biological agents through optimizing rice growing environment to improve yield showed great potential.

## 1 Introduction

As the world’s population grows, food production faces unprecedented challenges. By 2050, the world’s population will reach 9.6 billion, an increase of 34% from today (Tripathi et al., 2019). Pursuing sustainable agricultural production technologies to increase crop yields is critical to ensuring global food security. Traditional agricultural production has certain limitations. For example, large amounts of pesticides and chemical fertilizers are used during crop cultivation. This affects the quality of agricultural products and causes environmental pollution. Excessive chemical substances such as fertilizers and pesticides will seriously pollute water (Kumar et al., 2021), decline soil quality, and affect the ecological balance. In addition, excessive resource consumption is also a major disadvantage of the traditional agricultural model. Under this model, lower land, water, and energy use efficiency may lead to excessive consumption and waste of resources. In recent years, green agriculture has received widespread attention (Koohafkan et al., 2012; Li et al., 2021). Green agriculture focuses on increasing yields while considering the harmony between agricultural production and the natural environment. By applying more advanced production technologies and sustainable agricultural management methods, creating a high-yielding, efficient, healthy, and adaptable agricultural ecosystem can provide important scientific guarantees for solving global food security issues. With the increasing attention paid to sustainable agricultural development, the continuous innovation and optimization of crop production technology has become a research hotspot.

Rhizosphere soil contains diverse microorganisms whose importance to plant health and productivity cannot be underestimated. In the rhizosphere soil, plants interact with associated microorganisms through chemical signals produced in response to specific stimuli (Solomon et al., 2023). Many of the microbes in the rhizosphere defend plants against pathogen invasion and help acquire nutrients from the soil (Lee et al., 2021; Chepsergon and Moleleki, 2023). For example, *Lactobacillus* plays a crucial role in promoting plant growth by increasing soil phosphate solubilizing ability (Anzuay et al., 2013; Si et al., 2024) and stimulating root elongation (Limanska et al., 2013; Si et al., 2024). *Bacillus amyloliquefaciens*, an excellent biofertilizer and biocontrol agent, regulates plant cell growth and increases plant resistance to biotic and abiotic stresses (Luo et al., 2022). Lots of evidence showed that *Brevibacillus laterosporus* has the ability to contribute to fertility and to support plant health (Javed et al., 2020; Ruiu, 2020; Hamze and Ruiu, 2022; Wang et al., 2022). Therefore, using beneficial microorganisms to improve crop yield and conducting in-depth research on the influencing mechanisms are of great significance to promoting sustainable agricultural development. The compound microbial agent JZ used in this study contains a variety of beneficial bacteria, such as *Bacillus licheniformis, Bacillus amyloliquefaciens*, *Lactobacillus plantarum*, and *Lactobacillus casei*. This is different from previous studies that focus on the effects of a single bacterial species on plants. The regulation mechanism of this compound microbial agent on plants may be more complex.

In addition, a new type of biological agent MZ made from various plant monomer enzymes or plant extracts and chelated through two unique low-temperature aerobic fermentations has recently attracted the attention of many Chinese researchers. The main ingredients of this biological agent obtained by special processing of plant materials include papain, chamomile extract, bitter bark extract, etc., which has been applied to many crops, such as wheat (Sang et al., 2021), tea trees (You et al., 2021), and rice (Shao et al., 2022). Shao et al. (2022) found that the application of MZ increased the number of filled grains per panicle by 2.4 grains, the thousand-grain weight by 0.6 g, and the theoretical yield per 667 m^2^ by 63.5 kg, with a yield increase of 10.23% compared with the control. This means that MZ has excellent potential in increasing rice yield. Its application in agriculture is an effective means to promote production and increase income. Although MZ has shown potential in increasing rice yield, its effects on rice rhizosphere microbial diversity and soil metabolism have not been studied.

Based on previous studies, we speculated that using MZ and JZ may affect rhizosphere microecological environment and nutrient availability to promote rice production. The results of this study will provide a reference for achieving sustainable agriculture.

## 2 Materials and methods

### 2.1 Test site

This study was conducted in Shenzhen-Shanwei Special Cooperation Zone, Shanwei City (20°27′-23°28′N and 114°54′-116°13′E) in 2023. According to Shenzhen Climate Bulletin, the average annual precipitation in the Shenzhen-Shanwei Special Cooperation Zone was 1928.9 mm, the maximum daily rainfall was 238.7 mm, the maximum 1-h rainfall was 80.4 mm, and the annual average temperature was 23.9°C in 2023.

### 2.2 Experimental material

The rice variety Liliangyou8022, provided by Hunan Hybrid Rice Research Center, was used in this study. JZ was provided by Inner Mongolia Ketuo Microecological Technology Development Co., Ltd. The effective strains of JZ: *Bacillus licheniformis*, *Bacillus amyloliquefaciens*, *Lactobacillus plantarum*, and *Lactobacillus casei*; the number of effective viable bacteria≥2.0 × 10^8^ cfu·ml^−1^. MZ was provided by Heilongjiang Senqi Biotechnology Co., Ltd. The main ingredients of MZ are papain, chamomile extract, and bitter bark extract.

### 2.3 Experiment design

Rice seedlings were transplanted at the 4-leaf stage, with 2–3 plants per hole and a 19.98 × 26.64 cm density. The dosage of JZ per 667 m^2^ was 7 L aqueous solution (diluted with water to 20 times the undiluted original solution when used). JZ aqueous solution was evenly poured at the 4-leaf stage (2 days earlier than the day of transplanting), tillering stage, early heading stage, and full heading stage. The dosage of MZ per 667 m^2^ was 50 mL of undiluted original solution. MZ aqueous solution was sprayed on the leaves at the 4-leaf stage (2 days earlier than the day of transplanting), tillering stage, early heading stage, and full heading stage (50 mL of MZ was mixed with 15 L of water). This study set each treatment as a large plot (120 m^2^). There were three treatments, namely MZ, JZ, and CKZ (control). Sampling was done by randomly selecting three points in each treatment (with three replicates).

In this study, 300 kg·hm^−2^ of compound fertilizer (N, P, and K were all 15%) was used as base fertilizer; 225 kg·hm^−2^ of compound fertilizer and 75 kg·hm^−2^ of urea were used as tillering fertilizer; 37.5 kg·hm^−2^ of urea and 112.5 kg·hm^−2^ of potassium chloride were used as panicle fertilizer.

### 2.4 Determination of soil nutrient content and enzyme activity

Determination of soil nutrient content and enzyme activity was performed by South Subtropical Crop Research Institute, China Academy of Tropical Agricultural Sciences.

Soil from 0-10 cm layer was collected at the heading stage. pH, rapidly available potassium, available phosphorus, alkaline-hydrolysable nitrogen, total nitrogen, total phosphorus, total potassium, and organic matter content were determined based on the method of Bao (2000).

The determination of sucrase (S-SC) activity was described according to the method of the kit provided by Suzhou Grace Biotechnology Co., Ltd. S-SC catalyzes the degradation of sucrose to produce reducing sugars, which further react with 3,5-dinitrosalicylic acid to generate colored amino compounds with characteristic light absorption at 540 nm. Within a certain range, the increase rate of 540 nm light absorption is proportional to S-SC activity.

Catalase (S-CAT) activity was measured according to the method described in the kit provided by Beijing Solarbio Science & Technology Co., Ltd. S-CAT plays an important role in the H_2_O_2_ scavenging system. H_2_O_2_ has a characteristic absorption peak at 240 nm. By measuring the change in absorbance of the solution after reaction with soil at this wavelength, the level of S-CAT activity can be reflected.

Urease (S-UE) activity was measured according to the method described in the kit provided by Beijing Solarbio Science & Technology Co., Ltd. S-UE can hydrolyze urea to produce ammonia and carbonic acid. The indophenol blue colorimetric method was used to determine the NH_3_-N produced by S-UE hydrolyzes urea.

Acid phosphatase (S-ACP) activity was measured according to the method described in the kit provided by Beijing Solarbio Science & Technology Co., Ltd. In an acidic environment, S-ACP catalyzes the hydrolysis of disodium phenyl phosphate to produce phenol and disodium hydrogen phosphate. The S-ACP activity can be calculated by measuring the amount of phenol produced.

### 2.5 Determination of SPAD value and yield

The SPAD value was measured by SPAD-502Plus (Konica Minolta) at the heading stage. Rice yield was measured at the maturity stage.

### 2.6 Metabolome detection

Rhizosphere soil was collected at the heading stage, with 6 biological replicates for each treatment. Metabolome detection was performed by Shanghai Majorbio Bio-pharm Technology Co., Ltd.

#### 2.6.1 Sample treatment

Soil sample: 1,000 ± 5 mg sample and a 6 mm diameter grinding bead were placed in a 2 mL centrifuge tube. Then, 1,000 μL of extraction solution was added. After grinding with a grinder for 6 min (−10°C, 50 Hz), low-temperature ultrasonic extraction was performed for 30 min (5°C, 40 KHz). The sample was placed at −20°C for 30 min and then centrifuged for 15 min (13,000 g, 4°C). The supernatant was removed and dried with nitrogen gas. Subsequently, 120 μL of reconstitution solution (acetonitrile: water = 1:1) was added to reconstitute. After vortexing and mixing for 30 s, low-temperature ultrasonic extraction was performed for 5 min (5°C, 40KHz). Finally, the supernatant was used for analysis after centrifugation for 15 min (13,000 g, 4°C).

#### 2.6.2 LC–MS detection

LC–MS analysis was performed on a Thermo UHPLC-Q Exactive HF-X system.

Chromatographic conditions: the chromatographic column was ACQUITY UPLC HSS T3 (100 mm × 2.1 mm i.d., 1.8 μm; Waters, United States); mobile phase A was 95% water + 5% acetonitrile (containing 0.1% formic acid); mobile phase B was 47.5% acetonitrile + 47.5% isopropanol + 5% water (containing 0.1% formic acid); the injection volume was 3 μL and the column temperature was 40°C.

#### 2.6.3 Mass spectrometry conditions

The samples were ionized by electrospray, and the mass spectrum signals were collected using positive and negative ion scanning modes. The specific parameters were shown in Table 1.

**Table 1:** Mass spectrometry parameters.

Description	Parameter
Scan type (m/z)	70–1,050
Sheath gas flow rate (arb)	50
Aux gas flow rate (arb)	13
Heater temperature (°C)	425
Capillary temperature (°C)	325
Spray voltage (+) (V)	3,500
Spray voltage (−) (V)	−3,500
S-Lens RF Level	50
Normalized collision energy (eV)	20,40,60
Resolution (Full MS)	60,000
Resolution (MS^2^)	7,500

### 2.7 Microbial diversity detection

Rhizosphere soil was collected at the heading stage, with three biological replicates for each treatment. 16S rRNA sequencing and ITS sequencing were completed by Shanghai Majorbio Bio-pharm Technology Co., Ltd.

#### 2.7.1 Reagents and instruments

FastPure Soil DNA Isolation Kit (Shanghai Major Yuhua Bio-pharm Technology Co., Ltd); FastPfu Polymerase (TransGen Biotech Co., Ltd); T100 Thermal Cycler PCR instrument (BIO-RAD); JY600C electrophoresis apparatus (Beijing JUNYI DONGFANG Electrophoresis Co., Ltd).

#### 2.7.2 Sample DNA extraction

The total genomic DNA of the microbial community was extracted according to the instructions of the FastPure Soil DNA Isolation Kit (Shanghai Major Yuhua Bio-pharm Technology Co., Ltd). The quality of the extracted genomic DNA was checked by 1% agarose gel electrophoresis. The DNA concentration and purity were determined using NanoDrop2000 (Thermo Scientific).

#### 2.7.3 PCR amplification and sequencing

The primer design in this study was shown in Table 2.

**Table 2:** Primer design.

Sequencing region	Primer name	Primer sequences
338F_806R	338F	ACTCCTACGGGAGGCAGCAG
	806R	GGACTACHVGGGTWTCTAAT
ITS1F_ITS2R	ITS1F	CTTGGTCATTTAGAGGAAGTAA
	ITS2R	GCTGCGTTCTTCATCGATGC

The PCR reaction system for 338F_806R was: 5 × FastPfu Buffer (4 μL), 2.5 mM dNTPs (2 μL), 5 μM Forward Primer (0.8 μL), 5 μM Reverse Primer (0.8 μL), FastPfu Polymerase (0.4 μL), BSA (0.2 μL), and Template DNA (10 ng). ddH_2_O was added to make the system volume 20 μL. The amplification procedure was as follows: Pre-denaturation at 95°C for 3 min; 27 cycles (30 s at 95°C, 30 s at 55°C, 45 s at 72°C); then, the stable extension at 72°C for 10 min, and storage at 4°C (PCR instrument: ABI GeneAmp^®^ 9,700).

The PCR reaction system for ITS1F_ITS2R was: 10 × Buffer (2 μL), 2.5 mM dNTPs (2 μL), 5 μM Forward Primer (0.8 μL), 5 μM Reverse Primer (0.8 μL), rTaq Polymerase (0.2 μL), BSA (0.2 μL), and Template DNA (10 ng). ddH_2_O was added to make the system volume 20 μL. The amplification procedure was as follows: Pre-denaturation at 95°C for 3 min; 35 cycles (30 s at 95°C, 30 s at 55°C, 45 s at 72°C); then, the stable extension at 72°C for 10 min, and storage at 4°C (PCR instrument: ABI GeneAmp^®^ 9,700).

The sequencing was performed using the Illumina PE300 platform.

### 2.8 Statistical analysis

SPSS27 was used to perform independent samples *T*-test.

## 3 Result

### 3.1 Effect of MZ and JZ on soil nutrient content and enzyme activity

As shown in Supplementary Table S1, MZ and JZ significantly reduced soil pH. Rapidly available potassium and alkaline-hydrolysable nitrogen increased significantly, and available phosphorus significantly reduced after using MZ and JZ. The organic matter content was increased significantly after MZ treatment, while JZ had no significant effect on the organic matter content. In addition, both MZ and JZ could significantly increase the activities of S-CAT, S-UE, and S-ACP.

### 3.2 Effect of MZ and JZ on SPAD and yield

This study found that JZ and MZ positively regulated SPAD in rice leaves. The SPAD of JZ was significantly higher than that of CKZ. In addition, both JZ and MZ could significantly increase theoretical rice yield (Supplementary Table S2).

### 3.3 Metabolome of rice rhizosphere soil

#### 3.3.1 PCA, PLS-DA and OPLS-DA

**Figure 1:**
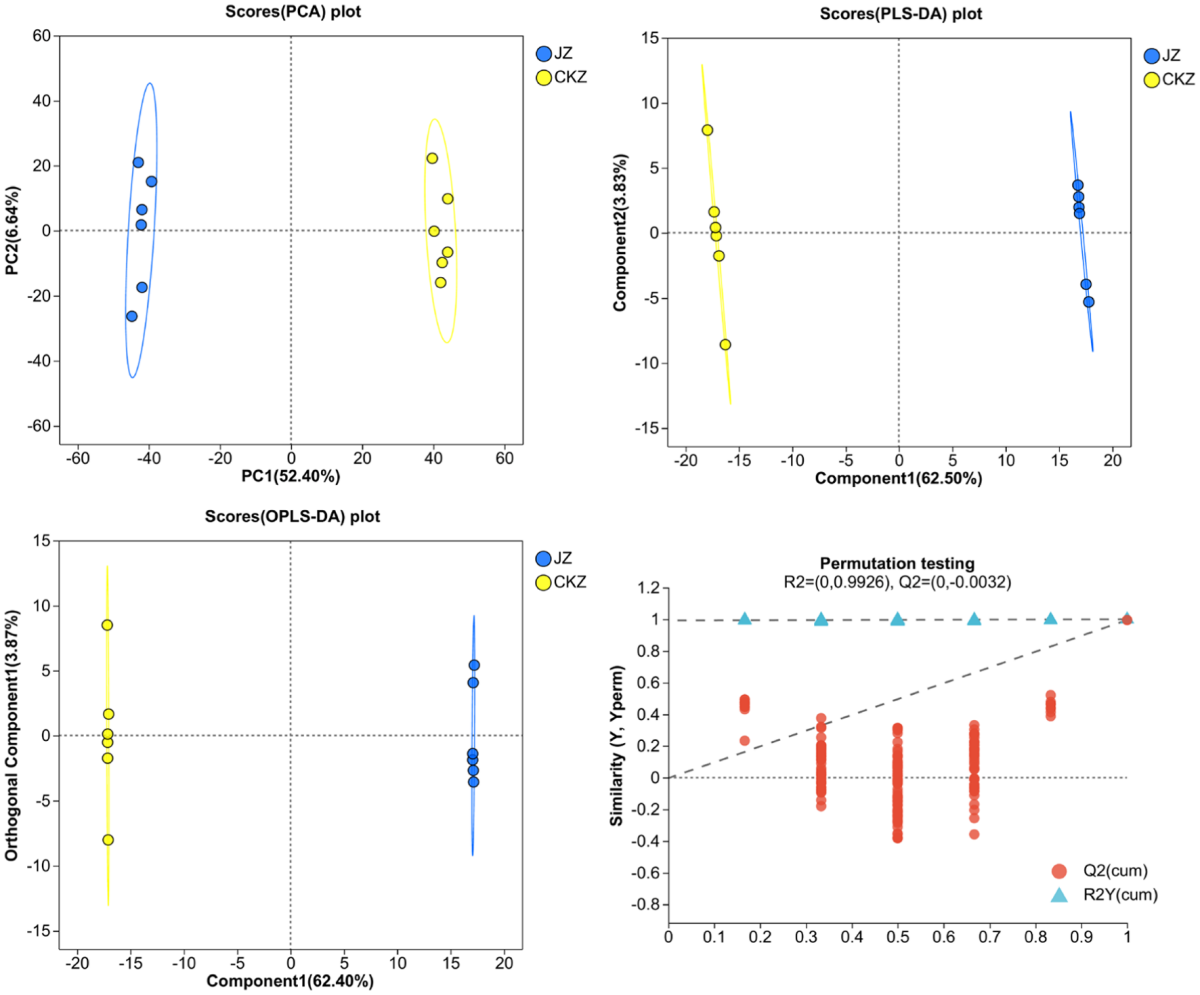
PCA, PLS-DA, OPLS-DA, and OPLS-DA permutation testing in CKZ/JZ in negative ion mode.

PCA, PLS-DA, and OPLS-DA results showed that the 6 samples of each treatment were distributed in the same area, and two treatments were separated (Figure 1; Supplementary Figures S1–S3). In addition, Q^2^ was greater than 0.5 in either positive or negative ion mode, indicating that the model had good prediction ability (Supplementary Tables S3–S6).

#### 3.3.2 Differential metabolite statistics and analysis

##### 3.3.2.1 In CKZ/JZ

**Figure 2:**
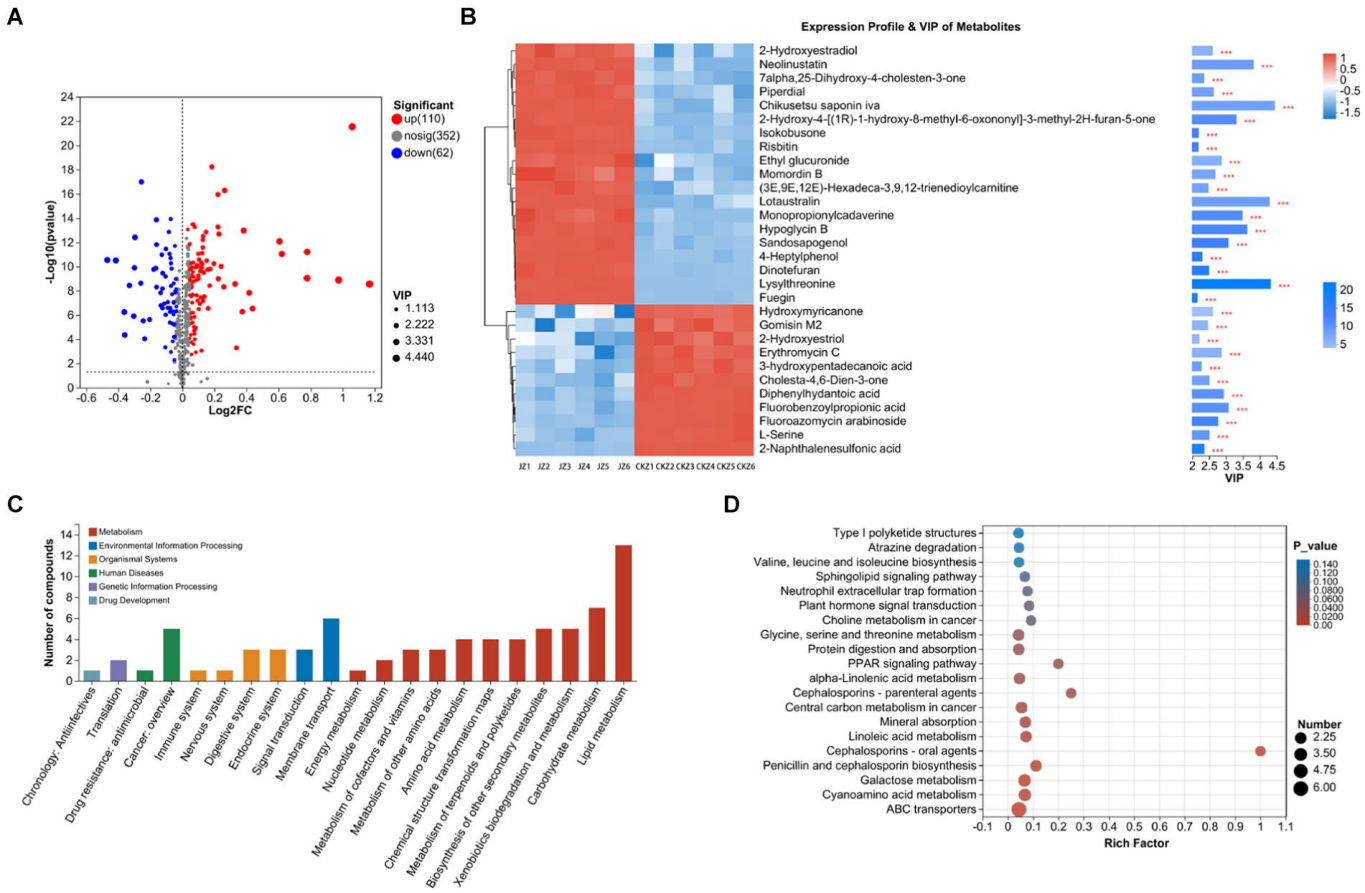
Statistics and analysis of differential metabolites in CKZ/JZ. **(A)** Volcano plot of differential metabolites. **(B)** VIP analysis. **(C)** KEGG annotation analysis. **(D)** KEGG enrichment analysis.

This study counted 172 differential metabolites, of which 110 were up-regulated, and 62 were down-regulated (Figure 2A). VIP analysis found that the differential metabolites with VIP greater than 4 were chikusetsu saponin iva, lotaustralin, and lysylthreonine, and these three metabolites were all up-regulated after the use of JZ (Figure 2B).

Further, KEGG annotation analysis found that the differential metabolites were mainly classified into lipid metabolism, carbohydrate metabolism, membrane transport, cancer: overview, biosynthesis of other secondary metabolites, and xenobiotics biodegradation and metabolism (Figure 2C). In addition, KEGG enrichment analysis found that DEGs were mainly enriched in the ABC transporters and biosynthesis of cofactors pathways (Figure 2D).

##### 3.3.2.2 In CKZ/MZ

As shown in Supplementary Figure S4A, 182 differential metabolites were detected in this study, of which 116 were up-regulated and 65 were down-regulated. Momordin B had the highest VIP value, followed by O-oxalylhomoserine (Supplementary Figure S4B).

KEGG annotation analysis found that differential metabolites were mainly classified into membrane transport, cancer: overview, amino acid metabolism, biosynthesis of other secondary metabolites, carbohydrate metabolism, lipid metabolism, xenobiotics biodegradation and metabolism, and digestive system (Supplementary Figure S5B). Further, KEGG enrichment analysis found that the differential metabolites were mainly enriched in the ABC transporters, galactose metabolism, nucleotide metabolism, and purine metabolism pathways (Supplementary Figure S5A).

### 3.4 16S rRNA sequencing

#### 3.4.1 Dilution curve and alpha diversity analysis

**Figure 3:**
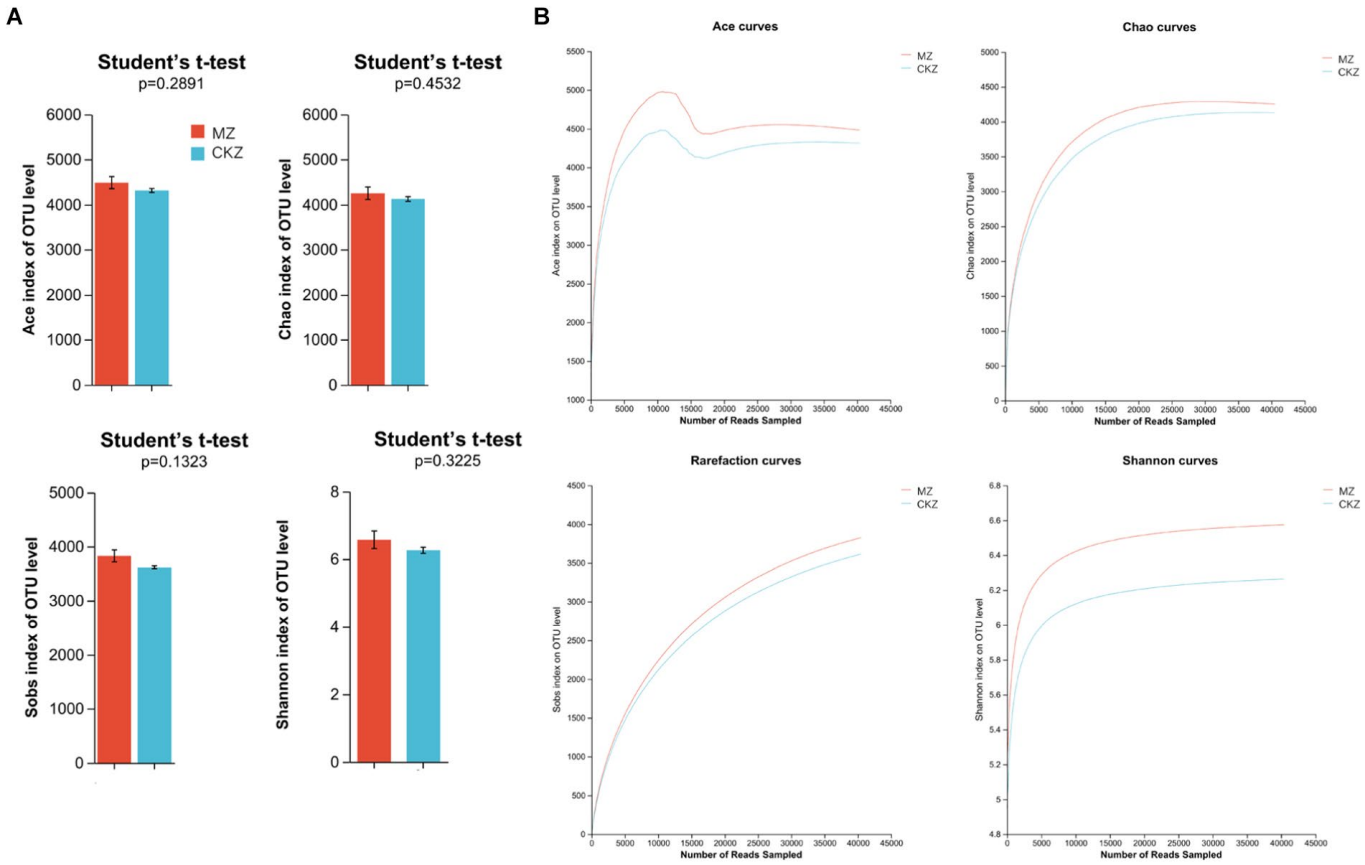
Dilution curve analysis and alpha diversity analysis in CKZ/MZ. **(A)** alpha diversity analysis; **(B)** dilution curve analysis.

The results of this study showed that the dilution curve tended to be flat; the sequencing data had basically reached saturation, and it could cover most species in the sample (Figure 3B; Supplementary Figure S6). This study used Ace, Chao, and Sobs indices to reflect community richness and Shannon index to reflect community diversity.

Alpha diversity analysis found no significant differences in community richness and community diversity between CKZ and JZ (Supplementary Table S7). Likewise, there was no significant change between CKZ and MZ (Figure 3A).

#### 3.4.2 Species composition analysis

##### 3.4.2.1 In CKZ/JZ

At the phylum level, the dominant microbial communities of CKZ and JZ treatments were *Firmicutes*, *Chloroflexi*, *Actinobacteriota*, *Proteobacteria*, and *Acidobacteriota*. Compared to CKZ, the percent of community abundance of *Firmicutes* and *Actinobacteriota* in JZ decreased, and that of *Chloroflexi* increased. At the genus level, the percent of community abundance of *Bacillus* in JZ increased, and that of unclassified_f__*Planococcaceae* decreased (Supplementary Figure S7).

##### 3.4.2.2 In CKZ/MZ

**Figure 4:**
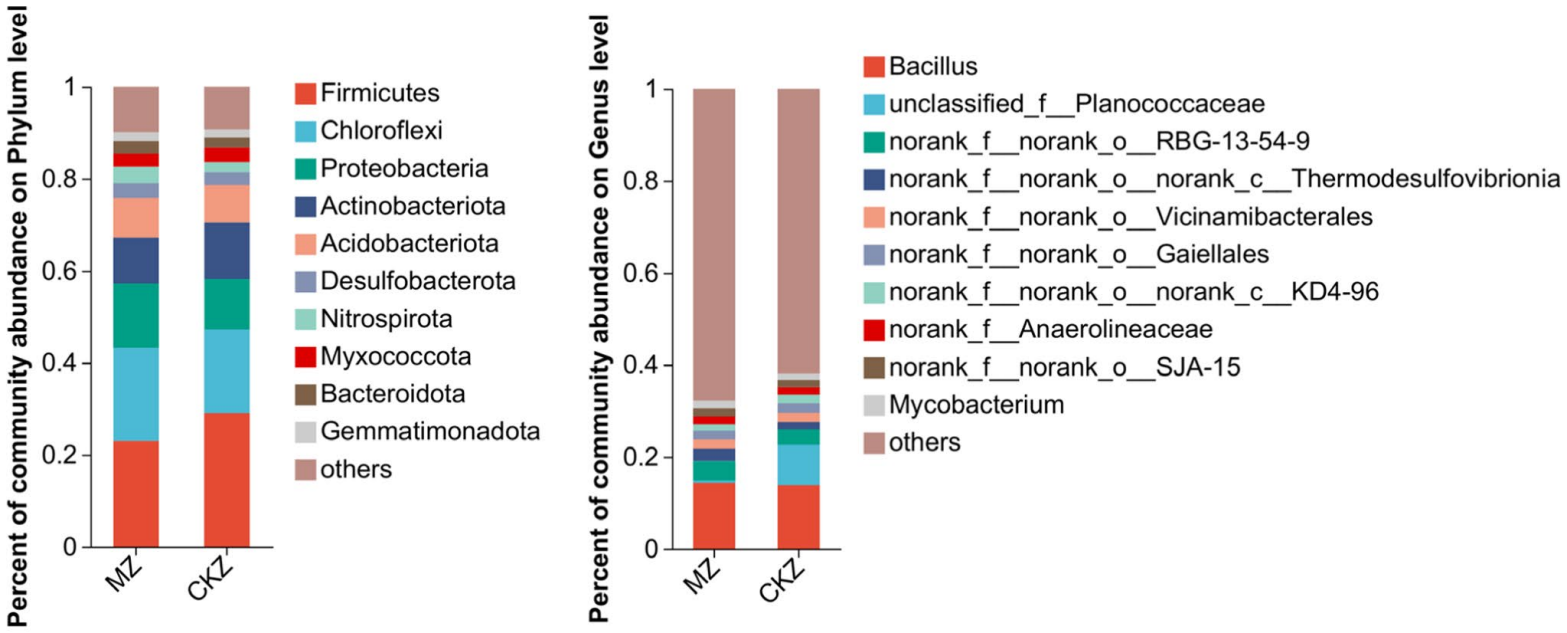
Species composition analysis in CKZ/MZ.

A total of 5 dominant microbial communities were detected at the phylum level, namely *Firmicutes*, *Chloroflexi*, *Proteobacteria*, *Actinobacteriota*, and *Acidobacteriota*. Compared with CKZ, the percent of community abundance of *Firmicutes* and *Actinobacteriota* decreased in MZ treatment; that of *Chloroflexi*, *Proteobacteria*, and *Acidobacteriota* increased. At the genus level, the percent of community abundance of unclassified_f__*Planococcaceae* decreased in MZ treatment compared with CKZ (Figure 4).

#### 3.4.3 Beta diversity analysis

**Figure 5:**
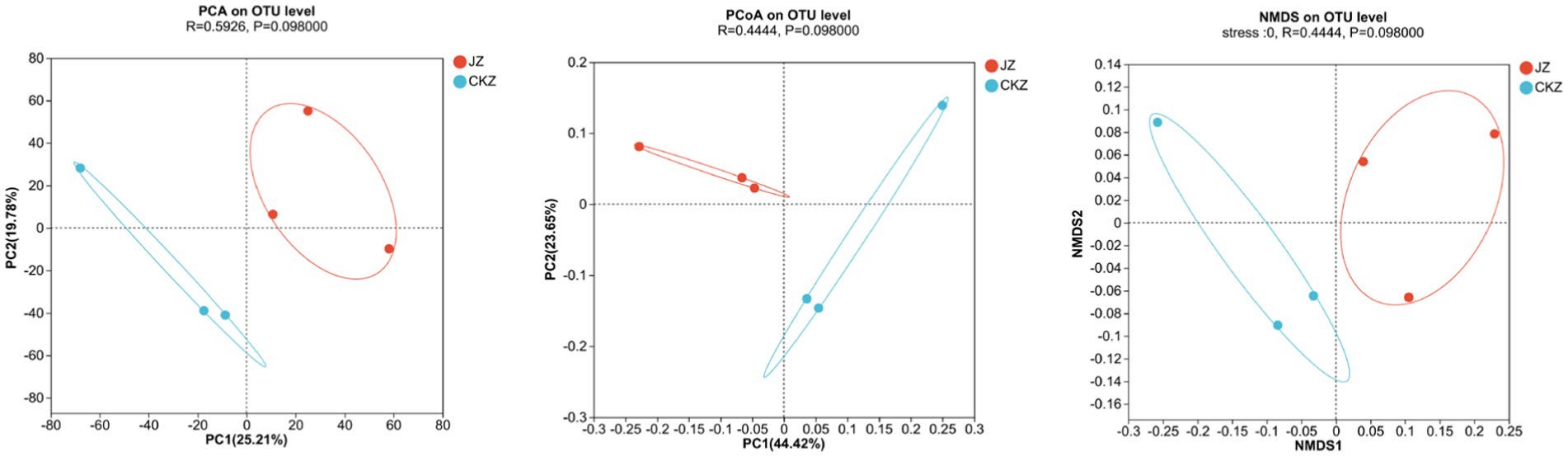
PCA, PCOA, and NMDS analysis in CKZ/JZ.

PCA, PCOA, and NMDS analysis showed a clear separation between the two treatments, indicating differences in microbial community composition between the two treatments (Figure 5; Supplementary Figure S8).

#### 3.4.4 Lefse multilevel species difference discriminant analysis

In CKZ/JZ, this study detected 182 differential communities at multiple levels, of which 96 were significantly enriched in JZ, and 86 were significantly enriched in CKZ (LDA threshold>2) (Supplementary Figure S9). In CKZ/MZ, 48 differential communities at multiple levels were obtained, of which 46 were significantly enriched in MZ, and 2 were significantly enriched in CKZ (LDA threshold>3) (Supplementary Table S8).

### 3.5 ITS sequencing

#### 3.5.1 Dilution curve and alpha diversity analysis

The results of this study showed that the dilution curve tended to be flat; the sequencing data had basically reached saturation, and it could cover most species in the sample (Supplementary Figures S10, S11B).

Alpha diversity analysis found no significant differences in community richness and community diversity between the CKZ and JZ (Supplementary Table S9). In addition, Ace, Sobs, and Chao indexes showed no significant changes after using MZ; the Shannon index decreased significantly after using MZ (Supplementary Figure S11A). These results indicated that the community diversity of MZ was lower compared to CKZ.

#### 3.5.2 Species composition analysis

##### 3.5.2.1 In CKZ/JZ

At the phylum level, the percent of community abundance of *Ascomycota* and *Basidiomycota* in JZ was higher compared with CKZ; that of unclassified_k__Fungi, *Rozellomycota*, and *Mortierellomycota* was lower. In addition, at the genus level, the percent of community abundance of *Sporidiobolus*, *Gibberella*, and *Penicillium* was higher in JZ; that of unclassified_k__Fungi, *Rozellomycota*, *Westerdykella*, *Saitozyma*, *Talaromyces*, and *Sordariomycetes* was lower compared to CKZ (Supplementary Tables S10, S11).

##### 3.5.2.2 In CKZ/MZ

A total of 6 dominant communities were detected at the phylum level, namely *Ascomycota*, unclassified_k__Fungi, *Rozellomycota*, *Basidiomycota*, *Mortierellomycota*, and *Chytridiomycota*. Compared with CKZ, the percent of community abundance of unclassified_k__Fungi, *Rozellomycota*, *Basidiomycota*, *Mortierellomycota*, and *Chytridiomycota* decreased in MZ treatment; that of *Ascomycota* increased. At the genus level, the percent of community abundance of *Gibberella*, *Penicillium*, and *Mortierella* increased in MZ treatment compared with CKZ; that of unclassified_k__Fungi, unclassified_p__*Rozellomycota*, unclassified_p__*Rozellomycota*, *Westerdykella*, *Saitozyma*, unclassified_c__*Sordariomycetes*, and *Talaromyces* decreased (Supplementaryt Figure S12).

#### 3.5.3 Lefse multilevel species difference discriminant analysis and beta diversity analysis

**Figure 6:**
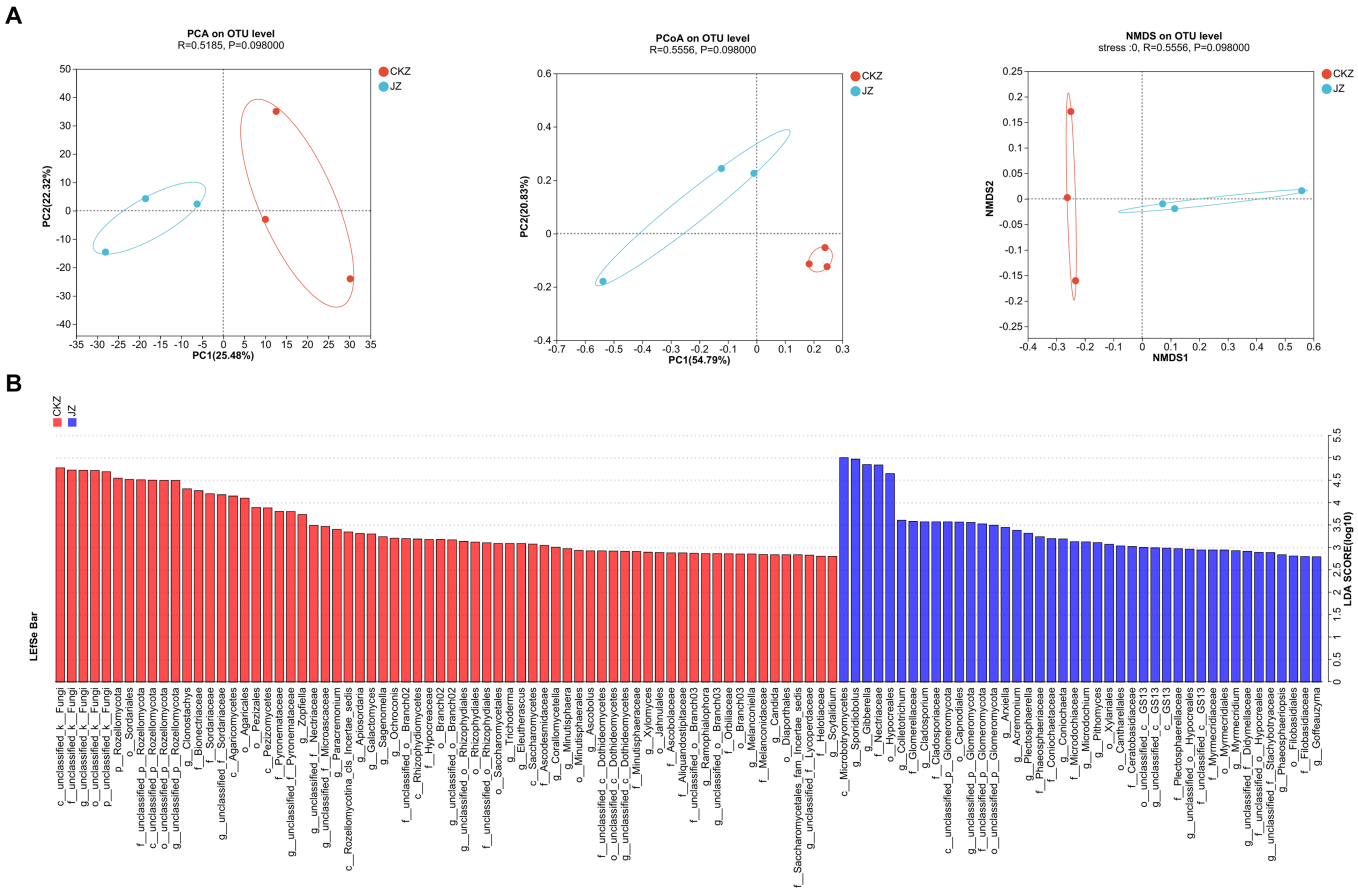
Lefse multilevel species difference discriminant analysis, PCA, PCOA, and NMDS analysis in CKZ/JZ. **(A)** PCA, PCOA, and NMDS analysis. **(B)** Lefse multilevel species difference discriminant analysis.

PCA, PCOA, and NMDS analysis results showed the differences in the community composition between the two treatments (Figure 6A; Supplementary Figure S13). The LDA discriminant histogram showed 110 differential communities in CKZ/JZ, of which 68 were significantly enriched in CKZ and 42 were significantly enriched in JZ (LDA threshold >2) (Figure 6B). In addition, 50 differential communities were screened in CKZ/MZ, of which 17 were significantly enriched in MZ, and 33 were significantly enriched in CKZ (LDA threshold >3) (Supplementary Figure S14).

## 4 Discussion

### 4.1 JZ and MZ affected the availability of nitrogen, phosphorus, and potassium

In this study, the rapidly available potassium and alkaline-hydrolysable nitrogen contents increased significantly after applying JZ and MZ. These are essential active nutrients in the soil and directly affect the growth and development of rice. The significant increase in S-UE activity in this study is an essential contributor to the increase in alkaline-hydrolysable nitrogen because this enzyme plays a role in urea degradation (Cordero et al., 2019). In addition, soil pH dropped significantly after JZ and MZ application. In this acidic soil environment, S-ACP plays an efficient role in organic P mineralization in soil (Huang et al., 2011). In this study, S-ACP activity significantly increased under JZ and MZ treatment conditions, which is beneficial for catalysing the decomposition of organic phosphorus. However, an unexpected result was that the available phosphorus in the JZ and MZ treatment was significantly reduced compared to that in CKZ. Here, we speculated that the decrease in available phosphorus content may be affected by the decrease in pH; in this soil environment, phosphoric acid may form a delayed effect state with calcium, iron or aluminum, reducing its effectiveness.

Furthermore, the results showed that using JZ and MZ significantly increased S-CAT activity. This antioxidant enzyme plays a role in antioxidant in the soil, indicating that JZ and MZ can prevent soil microorganisms and plants from oxidative damage.

### 4.2 JZ affected rice rhizosphere microecology

There are various ecological relationships between plants and microorganisms in the rhizosphere, such as competition, exploitation, commensalism, and mutualism, which affect plant growth and the overall function of the ecosystem (Solomon et al., 2023). *Acidobacteria* is one of the bacterial taxa in soil associated with the decomposition of soil organic matter (Banerjee et al., 2016; Kalam et al., 2020) and is structural and functional keystones in plant–soil microbiomes and agroecosystems (Kalam et al., 2020). In this study, c__*Acidobacteriae* and o__*Acidobacteriales* were significantly enriched in JZ treatment, indicating that JZ may play a role in the decomposition of soil organic matter. f__unclassified_p__*Cyanobacteria*, o__unclassified_p__*Cyanobacteria*, c__unclassified_p__*Cyanobacteria*, and g__unclassified_p__*Cyanobacteria* were also representatives significantly enriched in JZ treatment. According to report, *Cyanobacteria* play a major role in soil nitrogen fixation in agricultural fields and are widely used in rice fields (Joshi et al., 2020). Previous study showed that *Cyanobacteria* fix nitrogen up to 30%, produce some phytohormones, vitamins, amino acids, and organic acids, and increase rice productivity (Purwani et al., 2021). Therefore, the enrichment of *Cyanobacteria* in the JZ treatment may have contributed to the elevation of alkaline-hydrolysable nitrogen in this study, as it fixed nitrogen from the air and transformed it into the soil.

Additionally, ITS sequencing found that a total of 42 differential microbial communities, were significantly enriched in JZ, including g__*Cladosporium* and g__*Plectosphaerella*. *Cladosporium* species can secrete beneficial secondary metabolites to improve the ability of plants to adapt to new habitats and to sustain plant health and performance (Răut et al., 2021). The nematophagous fungus *Plectosphaerella cucumerina* has been reported to have the potential to against potato cyst nematodes populations by reducing field populations by up to 60% in trials (Atkins et al., 2003).

Based on the rhizosphere soil microbial community changes, this study conducted rhizosphere soil metabolome detection. 172 differential metabolites were detected between CKZ and JZ (Supplementary Table S12), indicating that rice rhizosphere soil metabolism changed after using JZ. In this study, the VIP values of the three up-regulated differential metabolites chikusetsu saponin iva, lotaustralin, and lysylthreonine were all greater than 4, ranking in the top 3 of all differential metabolites, indicating that these three metabolites had the highest contribution to the difference between the two treatments. Furthermore, this study found that these three metabolites had the strongest correlation with g__*Massilia* and g__*Plectosphaerella*. Previous studies found that *Massilia* sp. WF1 and *Massilia* sp. WG5 can degrade phenanthrene and repair contaminated soil (Lou et al., 2016; Gu et al., 2021; Xu et al., 2023). Interestingly, g__*Massilia* was significantly enriched in JZ. This revealed that JZ may potentially promote soil purification by enriching g__*Massilia*.

To further study the functions of differential metabolites in rhizosphere soil, this study conducted KEGG annotation on all differential metabolites and found that most metabolites were classified into lipid metabolism. Among these metabolites, jasmonic acid was significantly up-regulated after JZ application. Jasmonic acid is an endogenous growth regulatory substance, and exogenous application also has a regulatory effect on plants (Wang et al., 2020). A previous study found that jasmonic acid can contribute to rice resistance against *Magnaporthe oryzae* (Ma et al., 2022). Exogenous application of jasmonic acid triggers the defense mechanism of rice against *Rhizoctonia solani* Kühn (Younis et al., 2022). Therefore, up-regulation of jasmonic acid in the rhizosphere soil is beneficial to enhance rice’s disease resistance. In addition, JZ may also play a role in reducing pesticide residues. KEGG compound classification found that tricyclazole and dodemorph were significantly down-regulated; dinotefuran was significantly up-regulated. Since tricyclazole and dodemorph belong to fungicides, we speculated that using JZ changed the rhizosphere soil environment and promoted the degradation of fungicides. Dinotefuran is an insecticide, and its up-regulation may be due to the inhibition of dinotefuran degradation after JZ application. Research on the impact of JZ on pesticide residues in rice fields needs further exploration.

### 4.3 MZ affected rice rhizosphere microecology

The phylum *Bacteroidota* represents a group of bacteria, and these bacteria are dominant carbohydrate degraders in the soil (Larsbrink and McKee, 2020; Kruczyńska et al., 2023). Kruczyńska et al. (2023) found that the abundance of these beneficial microorganisms from the *Bacteroidota* community could potentially be connected with soil fertility, affecting crop yield (Kruczyńska et al., 2023). In this study, *Bacteroidota* was significantly enriched in the MZ treatment. In addition, at the genus level, *Bradyrhizobium* and *Pseudolabrys* were significantly enriched in MZ treatment. *Bradyrhizobium* performs essential biological functions in soil. Different representatives from the genus *Bradyrhizobium* were found to play essential roles in photosynthesis, symbiotic nitrogen fixation, and denitrification (Jones et al., 2016). Genus *Pseudolabrys* is also a beneficial biocontrol microorganism in plant rhizosphere soils (Fox et al., 2007; Tan et al., 2019).

Further, ITS sequencing found *Penicillium* was significantly enriched in MZ treatment at the genus level. Previous studies have reported that some *Penicillium* species can promote plant growth by production of PGP phytohormones, solubilization of minerals, or antagonism to phytopathogens (Wakelin et al., 2006; Nath et al., 2012; Radhakrishnan et al., 2013; Babu et al., 2015). Endophytic *Penicillium* spp. has been investigated for agricultural purposes to reduce the pollution of agricultural farms by chemical pesticides and heavy metals (Toghueo and Boyom, 2020).

In addition, 4-hydroxybutyric acid was up-regulated in the rhizosphere soil after application of MZ (Supplementary Table S13). 4-hydroxybutyrate together with threonine resulted in a significant increase in nitrogen and phosphorus content in root tissue under greenhouse conditions (Pantigoso et al., 2023).

## 5 Conclusion

The results of this study demonstrated that JZ and MZ can optimize the rice rhizosphere microecological environment by enriching beneficial microorganisms, adjusting rhizosphere soil metabolism, and improving nitrogen and potassium availability, effectively increasing rice yield, which provided a new agricultural management strategy. In the future, further research can be conducted into the long-term effects of JZ and MZ on rice growth under different environmental conditions, as well as how to optimize their use methods to maximize benefits in rice production.

## Data Availability

16S rRNA and ITS sequencing raw data were deposited in the Genome Sequence Archive in National Genomics Data Center, China National Center for Bioinformation/Beijing Institute of Genomics, Chinese Academy of Sciences (GSA: CRA020197 and CRA020200) that were publicly accessible at https://ngdc.cncb.ac.cn/gsa. Metabolome raw data was deposited in the OMIX, China National Center for Bioinformation/Beijing Institute of Genomics, Chinese Academy of Sciences: https://ngdc.cncb.ac.cn/omix with accession no. OMIX007778.
