# Supplementary material for: Effects of biological agents on rhizosphere microecological environment and nutrient availability for rice
Source: Front Microbiol. 2025 Jan 8;15:1447527. doi: 10.3389/fmicb.2024.1447527 (PMC11752750; doi:10.3389/fmicb.2024.1447527)
Supplement: Supplementary file 4 [file Data_Sheet_1.docx]

Table.S1 Effect of biological agents on soil nutrient content and enzyme activity

|  | CKZ | MZ | JZ |
| --- | --- | --- | --- |
| pH | 5.88±0.01a | 5.56±0.003c | 5.68±0.003b |
| Rapidly available potassium (mg·kg^-1^) | 26.42±1.15c | 65.14±0.16a | 59.24±0.72b |
| Available phosphorus (mg·kg^-1^) | 237.28±4.19a | 127.75±0.83c | 153.02±0.08b |
| Alkaline-hydrolysable nitrogen (mg·kg^-1^) | 131.60±1.25c | 185.12±1.16a | 157.92±1.62b |
| Organic matter (g·kg^-1^) | 43.94±0.30b | 50.72±0.27a | 43.58±0.40b |
| S-SC activity (U·g^-1^) | 49.90±0.87a | 42.80±1.36a | 48.90±3.30a |
| S-CAT activity (U·g^-1^) | 20.41±0.29c | 22.59±0.28a | 21.64±0.20b |
| S-UE activity (U·g^-1^) | 96.39±8.89c | 209.76±3.60a | 153.61±2.40b |
| S-ACP activity (U·g^-1^) | 27.33±0.88c | 46.43±1.19a | 41.27±0.23b |

Table.S2 Effect of biological agents on SPAD and yield

|  | SPAD | Theoretical yield (t·hm^2^) |
| --- | --- | --- |
| CKZ | 44.78±0.41b | 10.78±0.07c |
| JZ | 47.40±0.64a | 11.76±0.05b |
| MZ | 46.13±0.56ab | 12.94±0.03a |

Table.S3 OPLS-DA model parameters in positive ion mode in CKZ/JZ

| A | R^2^X | R^2^X (cum) | R^2^Y | R^2^Y (cum) | Q^2^ | Q^2^ (cum) |
| --- | --- | --- | --- | --- | --- | --- |
| p1 | 0.532 | 0.532 | 1 | 1 | 0.989 | 0.989 |
| sum | 0 | 0.578 | 0 | 1 | 0 | 0.99 |
| o1 | 0.0457 | 0.578 | 0.0002 | 0.0002 | 0.0004 | 0.0004 |

Table.S4 OPLS-DA model parameters in negative ion mode in CKZ/JZ

| A | R^2^X | R^2^X (cum) | R^2^Y | R^2^Y (cum) | Q^2^ | Q^2^ (cum) |
| --- | --- | --- | --- | --- | --- | --- |
| sum | 0 | 0.663 | 0 | 1 | 0 | 0.995 |
| o1 | 0.0387 | 0.663 | 0.0007 | 0.0007 | 0.0003 | 0.0003 |
| p1 | 0.624 | 0.624 | 0.999 | 0.999 | 0.994 | 0.994 |

Table.S5 Model parameters in positive ion mode in CKZ/MZ

| A | R^2^x | R^2^x(cum) | R^2^y | R^2^y(cum) | Q^2^ | Q^2^(cum) |
| --- | --- | --- | --- | --- | --- | --- |
| p1 | 0.675 | 0.675 | 1 | 1 | 0.997 | 0.997 |
| o1 | 0.0337 | 0.709 | 0.0001 | 0.0001 | 0.0001 | 0.0001 |
| sum | 0 | 0.709 | 0 | 1 | 0 | 0.997 |

Table.S6 Model parameters in negative ion mode in CKZ/MZ

| A | R^2^x | R^2^x(cum) | R^2^y | R^2^y(cum) | Q^2^ | Q^2^(cum) |
| --- | --- | --- | --- | --- | --- | --- |
| p1 | 0.734 | 0.734 | 1 | 1 | 0.998 | 0.998 |
| o1 | 0.0296 | 0.764 | 0.0002 | 0.0002 | 0.0001 | 0.0001 |
| sum | 0 | 0.764 | 0 | 1 | 0 | 0.998 |

Table.S7 Index difference test in CKZ/JZ

| Estimators | CKZ-Mean | CKZ-Sd | JZ-Mean | JZ-Sd | P_value(CKZ-JZ) | P_adjust(CKZ-JZ) |
| --- | --- | --- | --- | --- | --- | --- |
| Ace | 4313.1 | 74.235 | 4237.9 | 236.17 | 0.6267 | 0.8626 |
| Chao | 4128.5 | 89.865 | 4062.8 | 237.38 | 0.6773 | 0.8626 |
| Shannon | 6.2628 | 0.16358 | 6.3634 | 0.22912 | 0.5696 | 0.8626 |
| Sobs | 3614.7 | 49.136 | 3558 | 249.18 | 0.7188 | 0.8626 |

Table.S9 Index difference test in CKZ/JZ

| Estimators | CKZ-Mean | CKZ-Sd | JZ-Mean | JZ-Sd | P_value(CKZ-JZ) | P_adjust(CKZ-JZ) |
| --- | --- | --- | --- | --- | --- | --- |
| Ace | 740.65 | 28.334 | 665.06 | 226.33 | 0.5967 | 0.6007 |
| Chao | 740.91 | 28.86 | 664.56 | 231.23 | 0.6007 | 0.6007 |
| Shannon | 4.605 | 0.047261 | 3.5271 | 1.3513 | 0.2395 | 0.5873 |
| Sobs | 727.33 | 25.968 | 646.67 | 223 | 0.5674 | 0.6007 |

Table.S10 Community analysis at the phylum level in CKZ/JZ

| OTU ID | CKZ | JZ |
| --- | --- | --- |
| d__Eukaryota; k__Fungi; p__Ascomycota | 0.57362 | 0.607398 |
| d__Eukaryota; k__Fungi; p__Basidiomycota | 0.086093 | 0.262395 |
| d__Eukaryota; k__Fungi; p__unclassified_k__Fungi | 0.178441 | 0.063024 |
| d__Eukaryota; k__Fungi; p__Rozellomycota | 0.086372 | 0.015324 |
| d__Eukaryota; k__Fungi; p__Mortierellomycota | 0.044868 | 0.022343 |
| d__Eukaryota; k__Fungi; p__Chytridiomycota | 0.02877 | 0.027768 |
| d__Eukaryota; k__Fungi; p__Glomeromycota | 0.000511 | 0.001099 |
| d__Eukaryota; k__Fungi; p__Zoopagomycota | 0.000385 | 0.000413 |
| d__Eukaryota; k__Fungi; p__Blastocladiomycota | 0.000692 | 0 |
| d__Eukaryota; k__Fungi; p__Monoblepharomycota | 0.0001 | 9.08E-05 |
| others | 0.000147 | 0.000144 |

Table.S11 Community analysis at the genus level in CKZ/JZ

| OTU ID | CKZ | JZ |
| --- | --- | --- |
| d__Eukaryota; k__Fungi; p__unclassified_k__Fungi; c__unclassified_k__Fungi; o__unclassified_k__Fungi; f__unclassified_k__Fungi; g__unclassified_k__Fungi | 0.178441 | 0.063024 |
| d__Eukaryota; k__Fungi; p__Basidiomycota; c__Microbotryomycetes; o__Sporidiobolales; f__Sporidiobolaceae; g__Sporidiobolus | 0.002781 | 0.212942 |
| d__Eukaryota; k__Fungi; p__Ascomycota; c__Sordariomycetes; o__Hypocreales; f__Nectriaceae; g__Gibberella | 0.013176 | 0.158485 |
| d__Eukaryota; k__Fungi; p__Ascomycota; c__Eurotiomycetes; o__Eurotiales; f__Aspergillaceae; g__Penicillium | 0.024598 | 0.075125 |
| d__Eukaryota; k__Fungi; p__Rozellomycota; c__unclassified_p__Rozellomycota; o__unclassified_p__Rozellomycota; f__unclassified_p__Rozellomycota; g__unclassified_p__Rozellomycota | 0.080641 | 0.01405 |
| d__Eukaryota; k__Fungi; p__Ascomycota; c__Dothideomycetes; o__Pleosporales; f__Sporormiaceae; g__Westerdykella | 0.046816 | 0.039127 |
| d__Eukaryota; k__Fungi; p__Basidiomycota; c__Tremellomycetes; o__Tremellales; f__Trimorphomycetaceae; g__Saitozyma | 0.045444 | 0.038166 |
| d__Eukaryota; k__Fungi; p__Ascomycota; c__Eurotiomycetes; o__Eurotiales; f__Trichocomaceae; g__Talaromyces | 0.043512 | 0.027176 |
| d__Eukaryota; k__Fungi; p__Ascomycota; c__Sordariomycetes; o__unclassified_c__Sordariomycetes; f__unclassified_c__Sordariomycetes; g__unclassified_c__Sordariomycetes | 0.042046 | 0.027887 |
| d__Eukaryota; k__Fungi; p__Chytridiomycota; c__unclassified_p__Chytridiomycota; o__unclassified_p__Chytridiomycota; f__unclassified_p__Chytridiomycota; g__unclassified_p__Chytridiomycota | 0.028281 | 0.027135 |
| others | 0.494264 | 0.316884 |


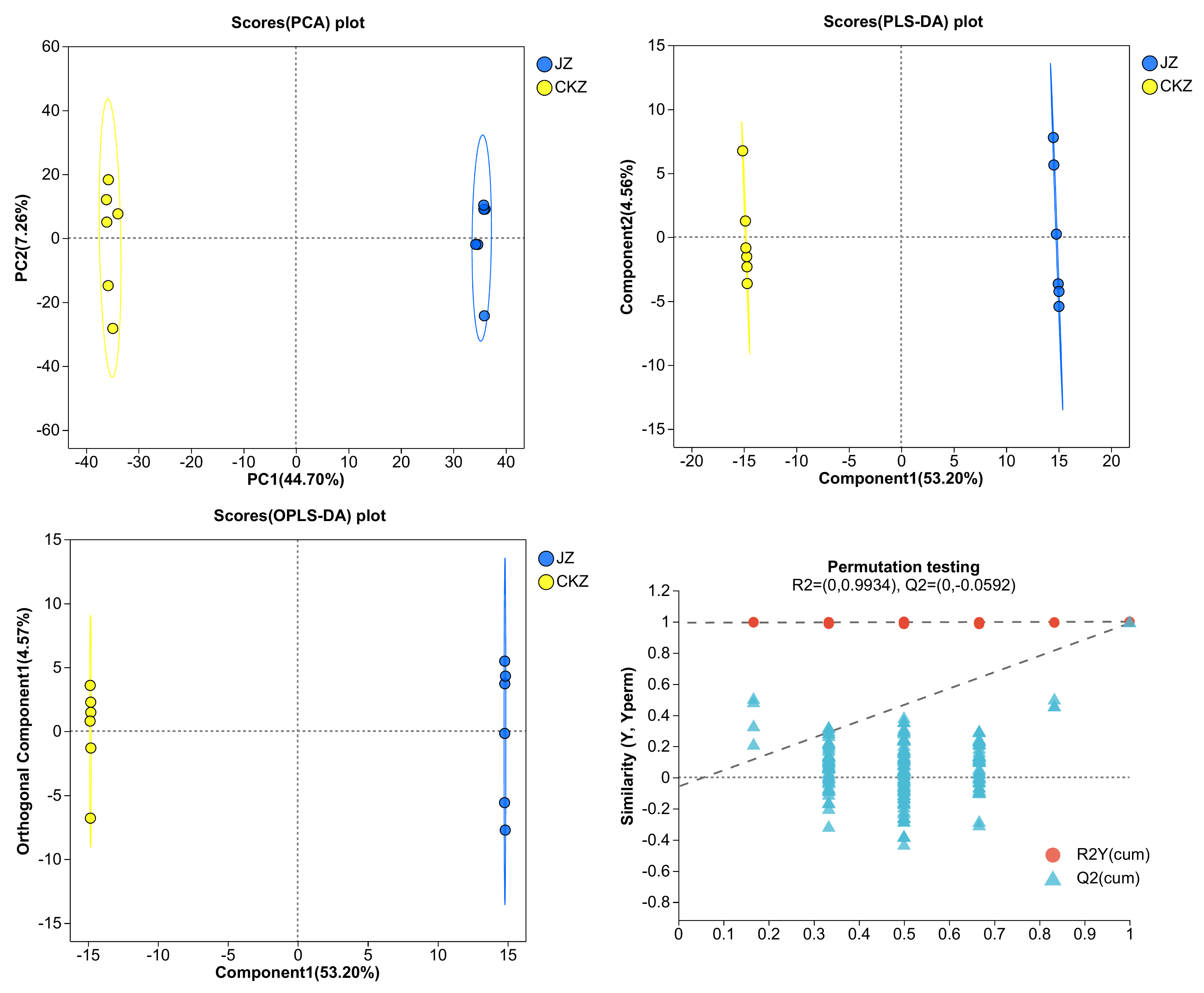


Fig.S1 PCA, PLS-DA, OPLS-DA analysis, and OPLS-DA permutation testing in CKZ/JZ in positive ion mode.


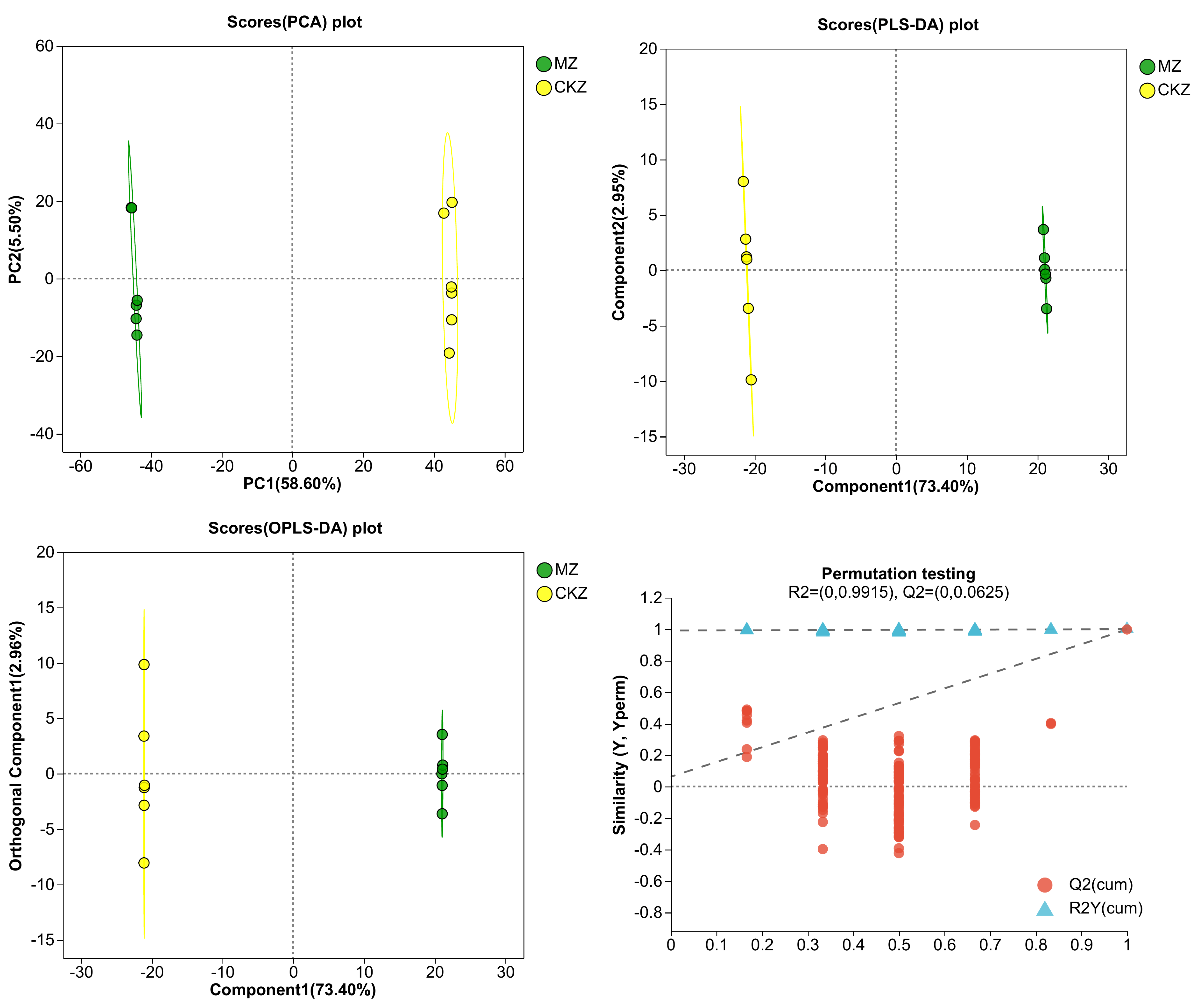


Fig.S2 PCA, PLS-DA, OPLS-DA analysis, and OPLS-DA permutation testing in CKZ/MZ in negative ion mode.


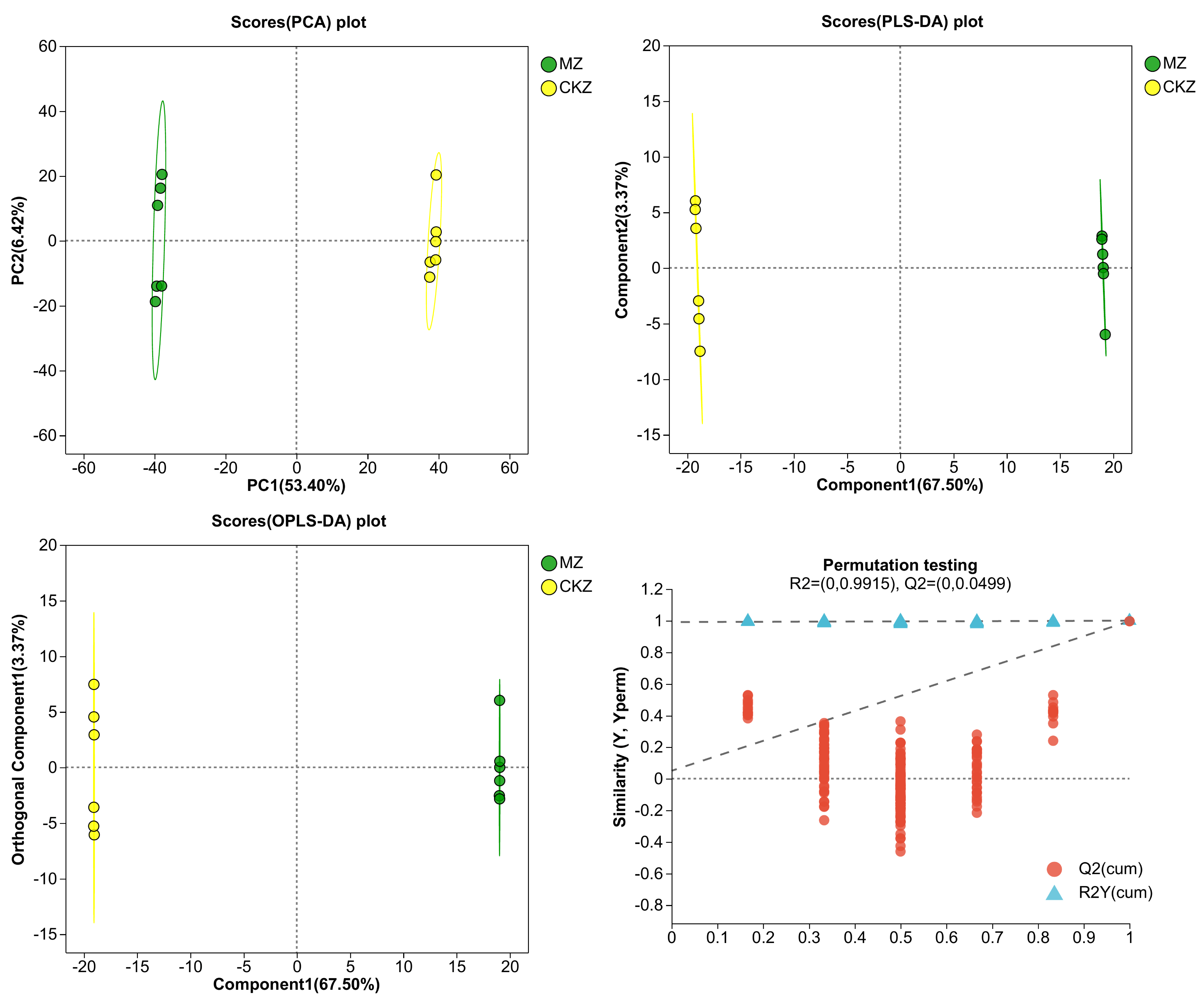


Fig.S3 PCA, PLS-DA, OPLS-DA analysis, and OPLS-DA permutation testing in CKZ/MZ in positive ion mode.


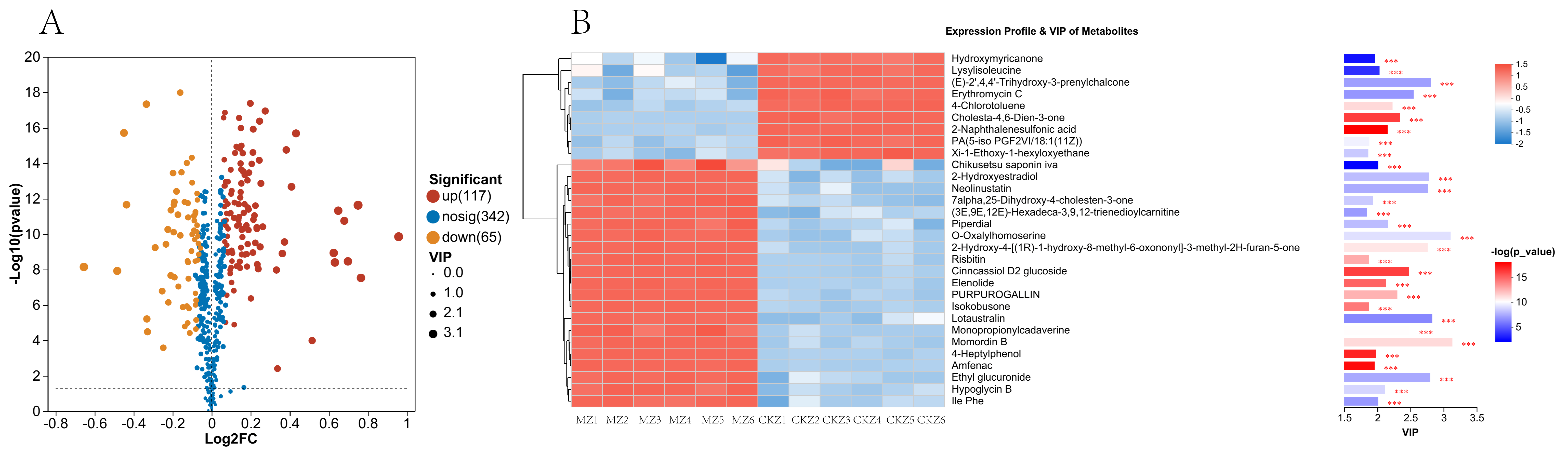


Fig.S4 Differential metabolite volcano plot, cluster analysis, and VIP analysis in CKZ/MZ. A: differential metabolite volcano plot; B: VIP analysis


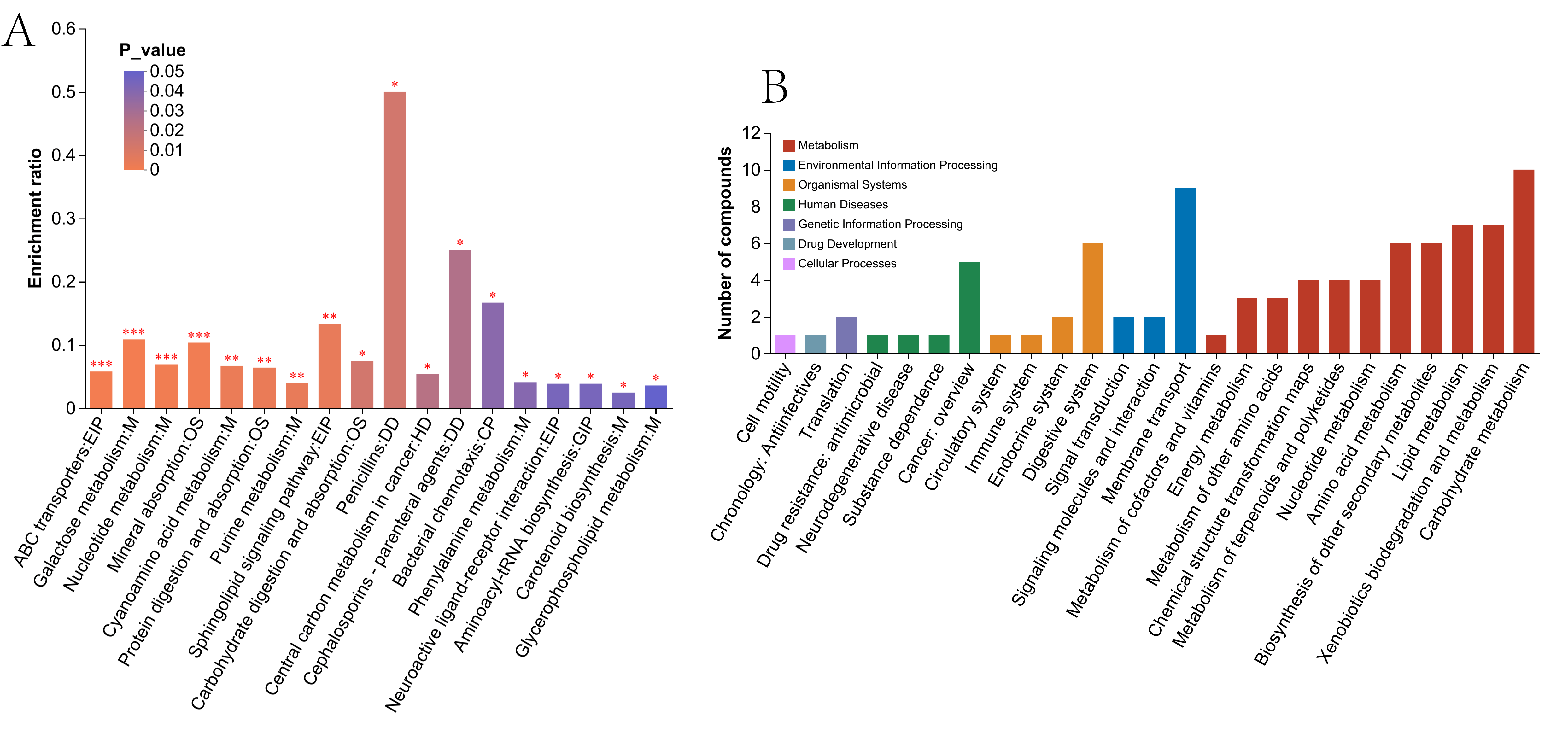


Fig.S5 KEGG annotation and enrichment analysis in CKZ/MZ. A: KEGG enrichment analysis; B: KEGG annotation analysis


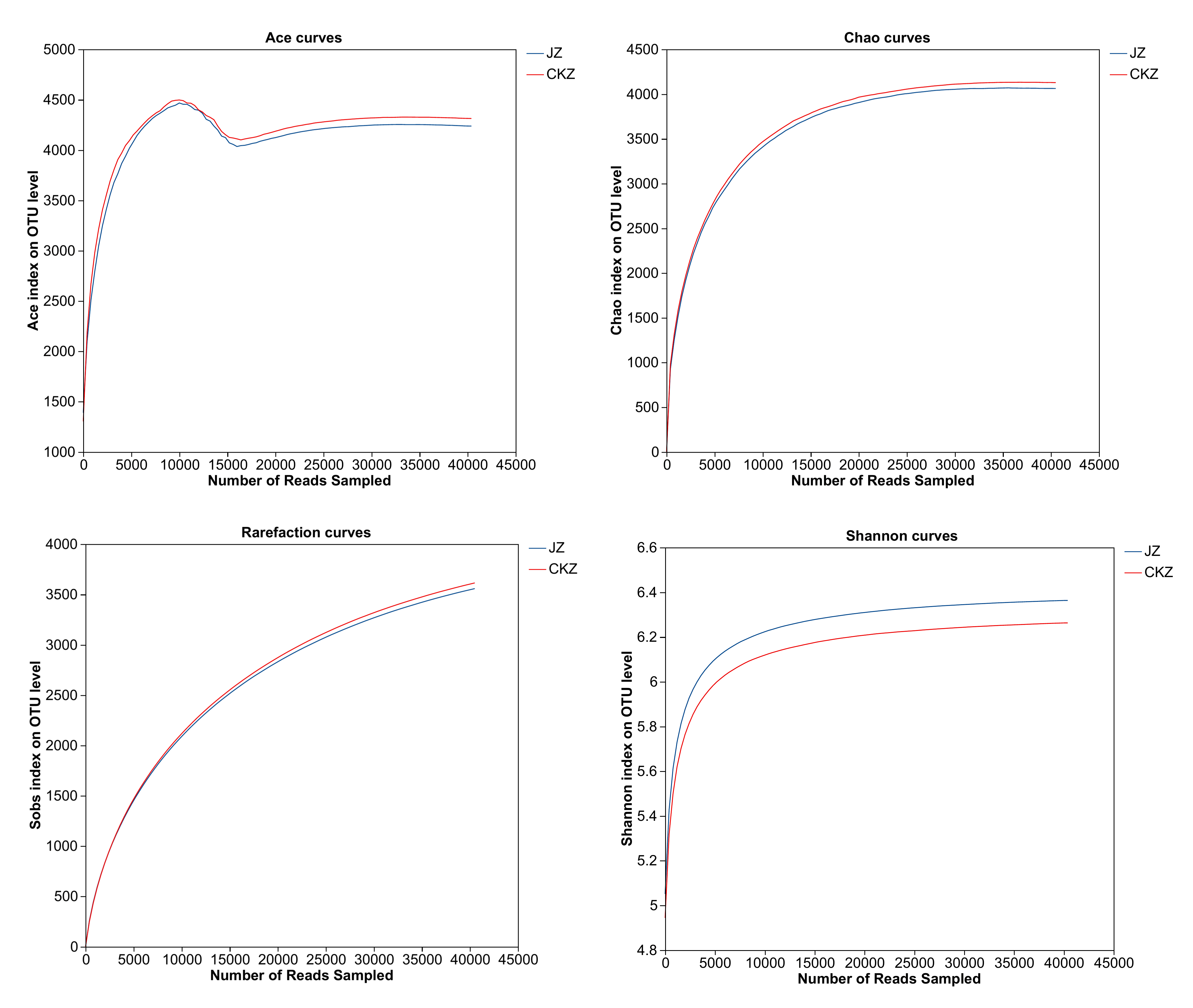


Fig.S6 Dilution curve analysis in CKZ/JZ.


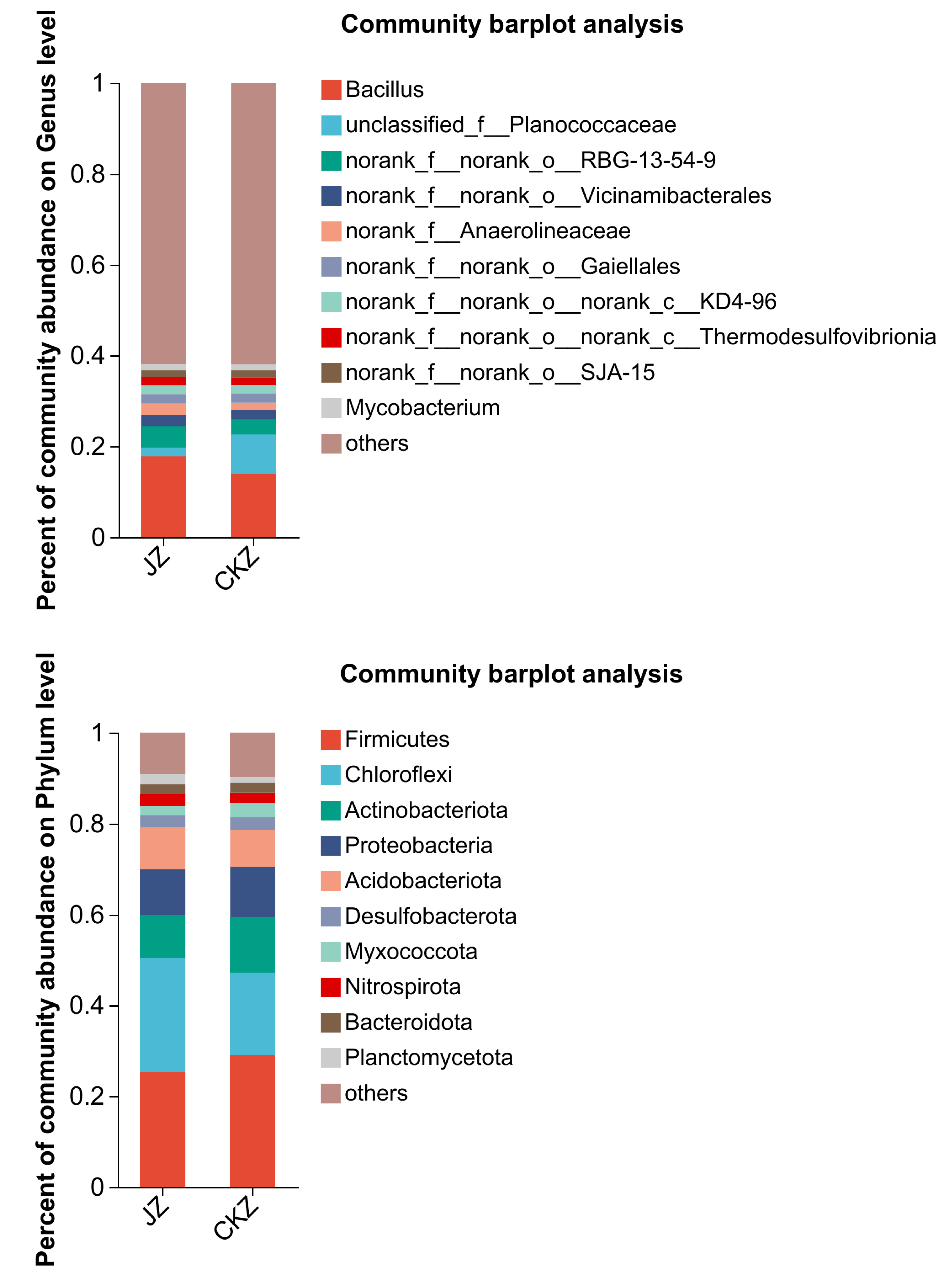


Fig.S7 Community analysis bar chart in CKZ/JZ.


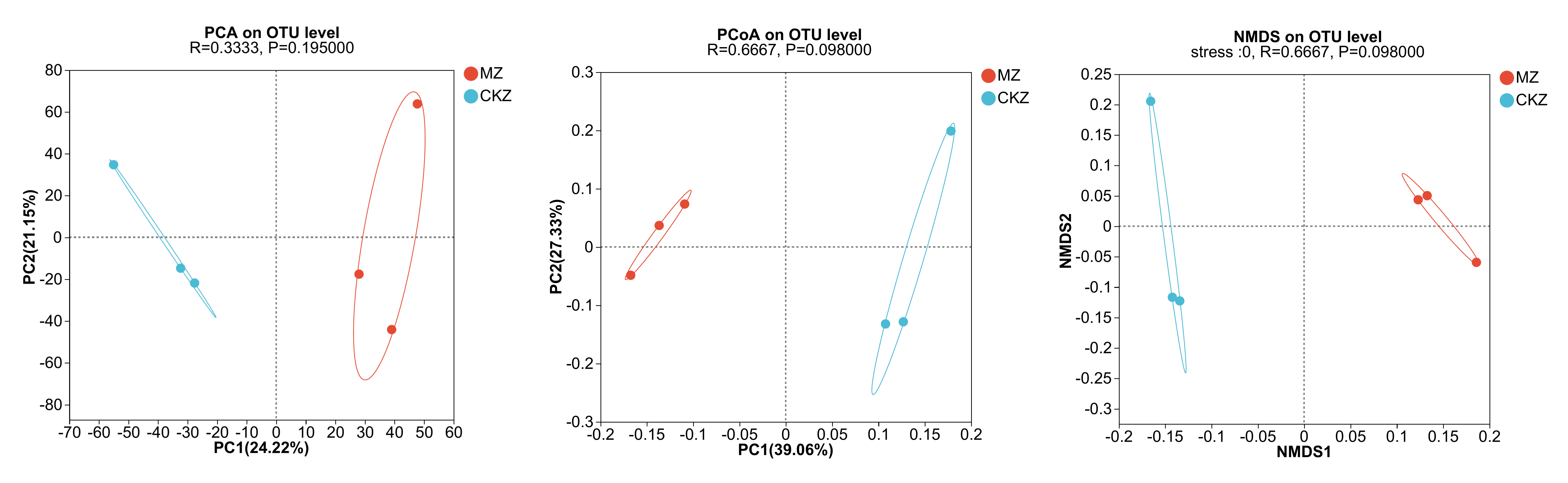


Fig.S8 PCA, PCOA, and NMDS analysis in CKZ/MZ.


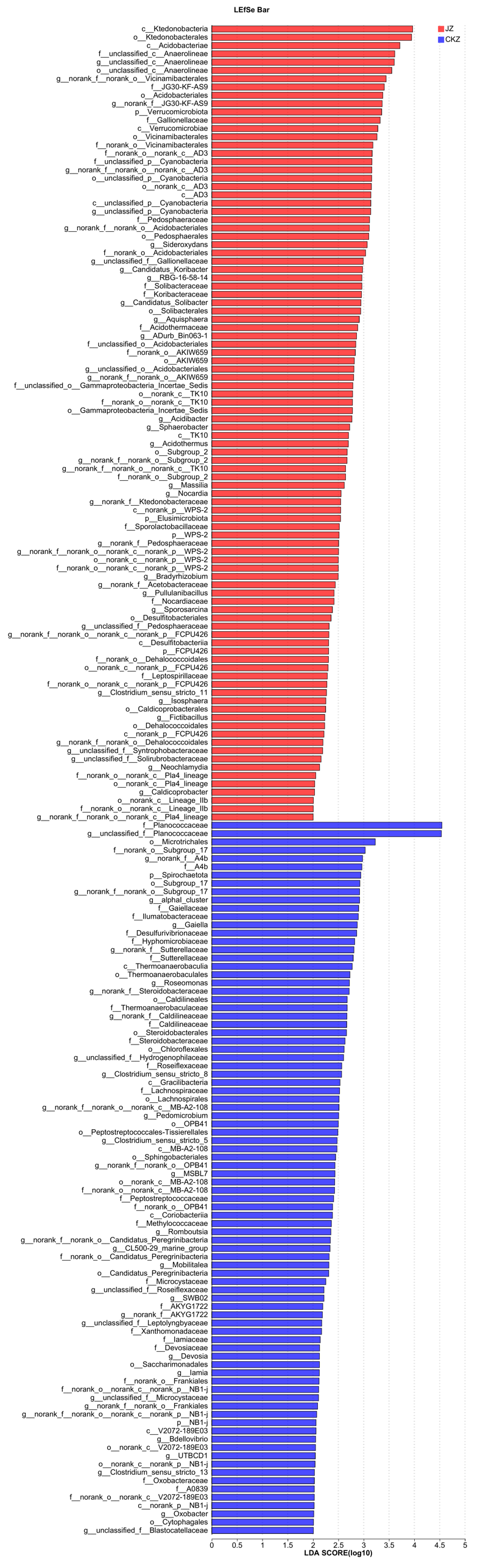


Fig.S9 Lefse multilevel species difference discriminant analysis in CKZ/JZ


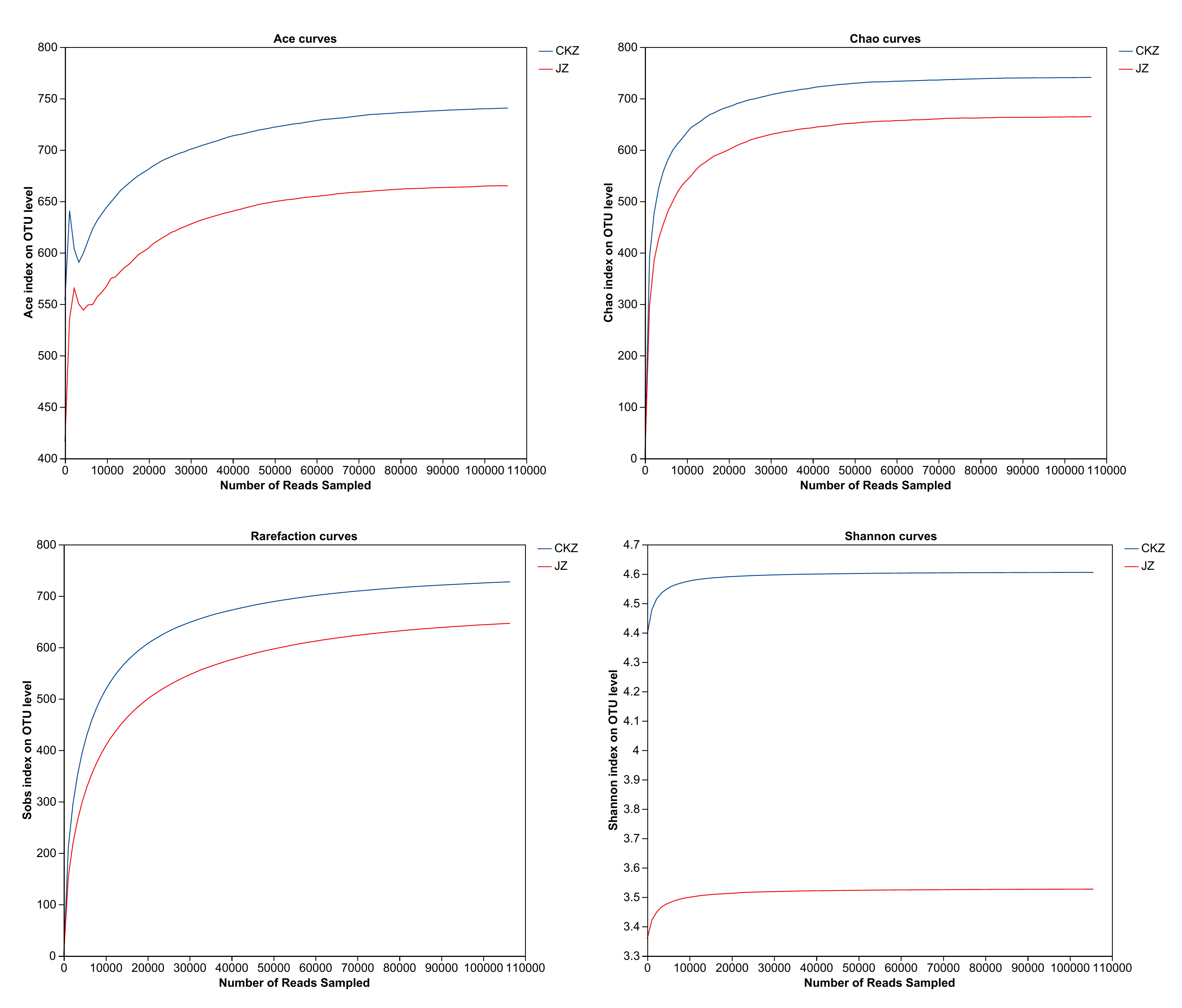


Fig.S10 Dilution curve analysis in CKZ/JZ


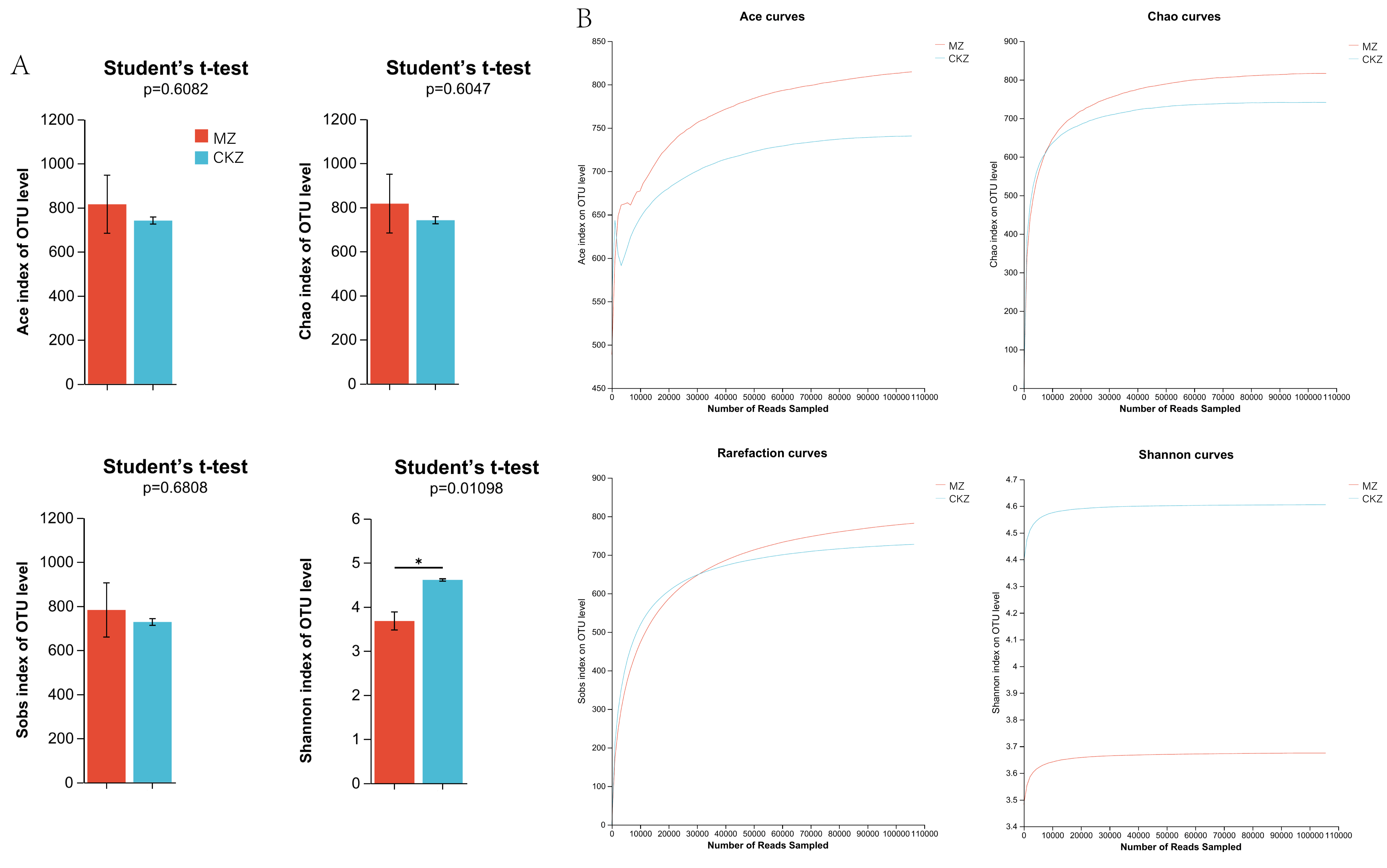
Fig.S11 Dilution curve analysis and alpha diversity analysis in CKZ/MZ. A: alpha diversity analysis; B: dilution curve analysis


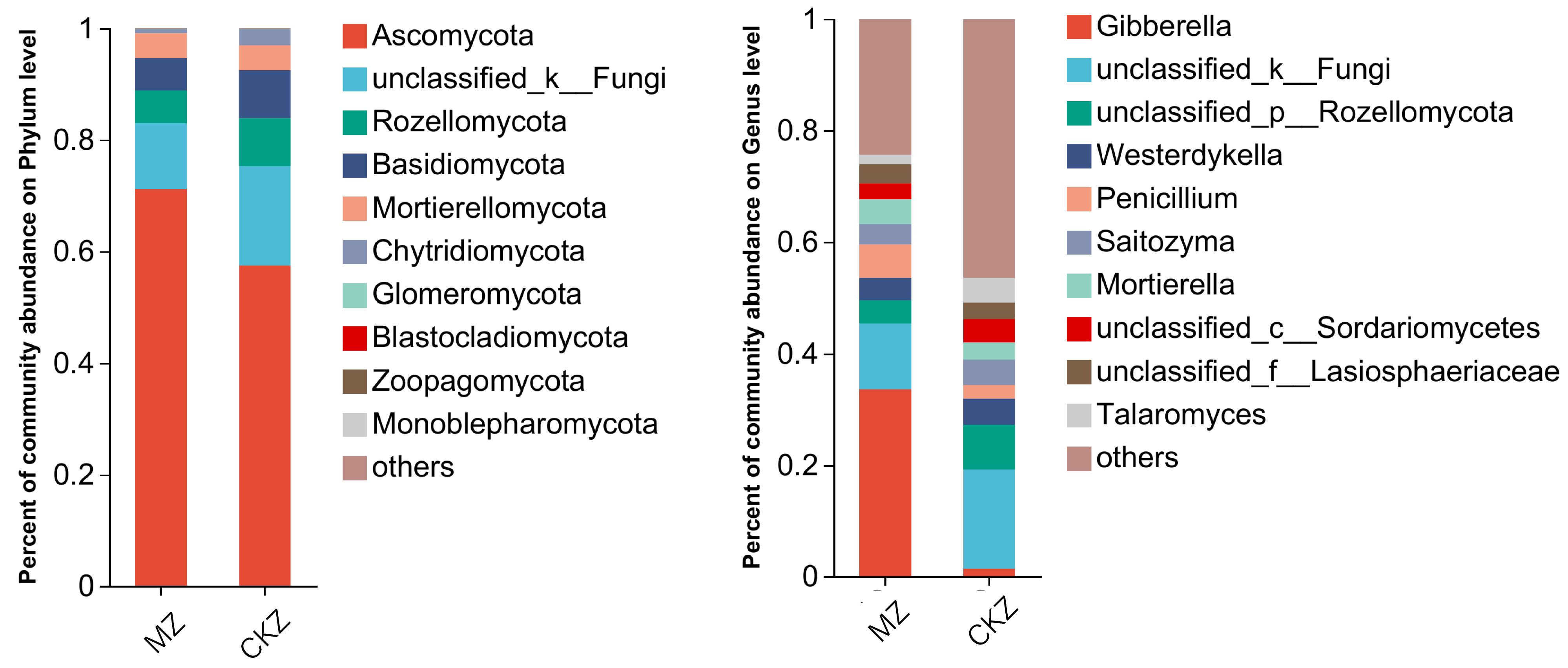


Fig.S12 Species composition analysis in CKZ/MZ


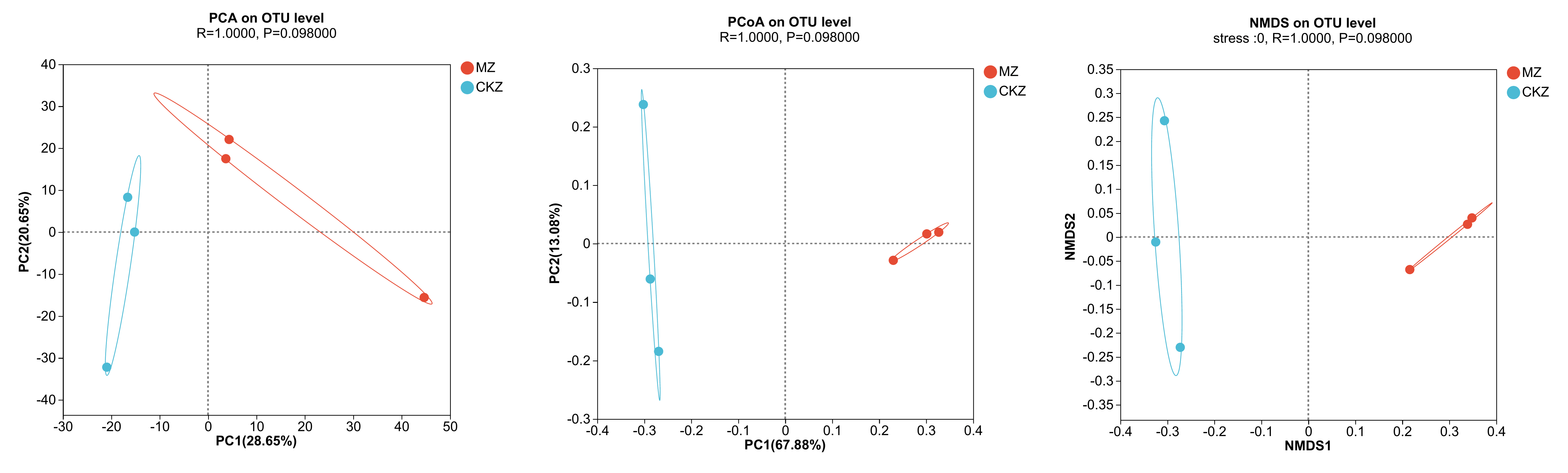


Fig.S13 PCA, PCOA, and NMDS analysis in CKZ/MZ


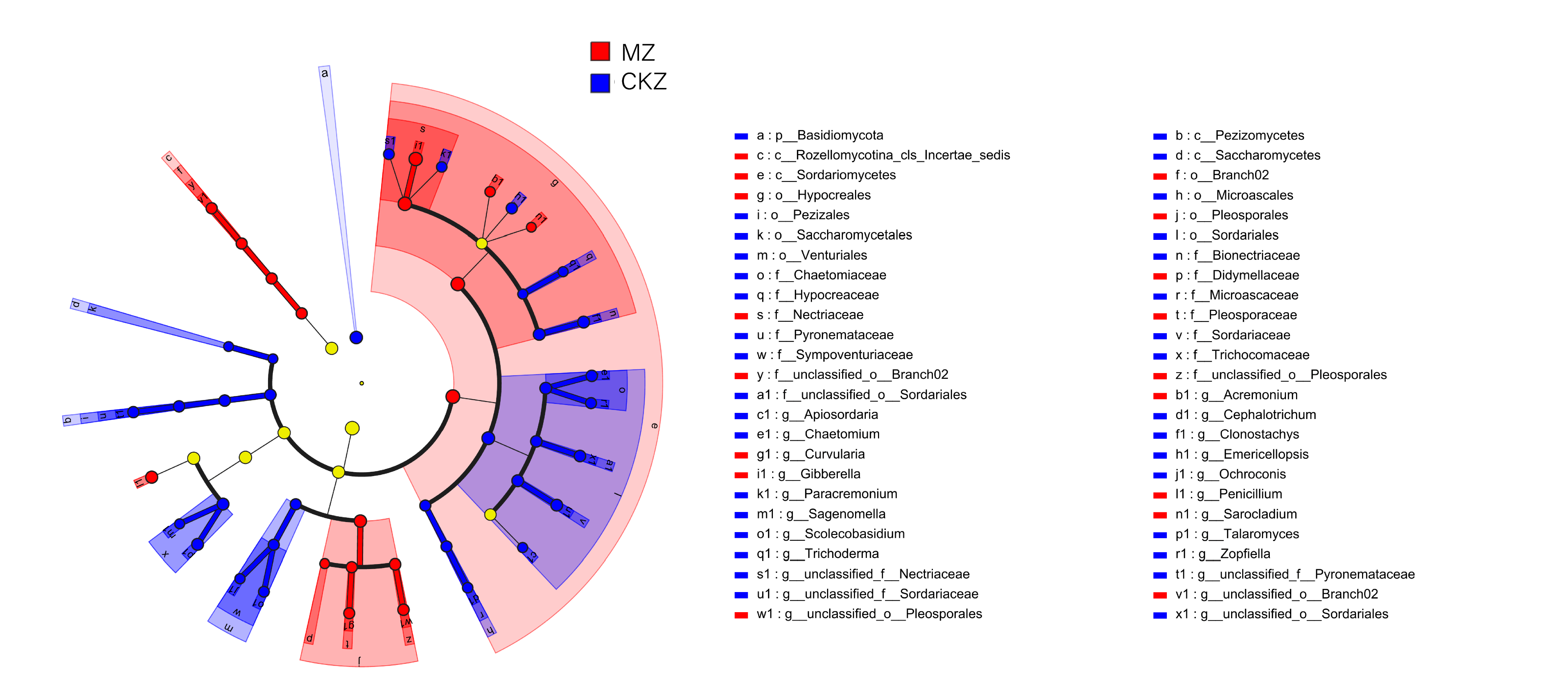


Fig.S14 Lefse multilevel species difference discriminant analysis in CKZ/MZ
